# Synthesis, Properties and Application of Novel 2-Substituted Benzothiazole-Based Oxime Esters

**DOI:** 10.3390/ma19030558

**Published:** 2026-01-30

**Authors:** Monika Dzwonkowska-Zarzycka, Alicja Balcerak-Woźniak, Janina Kabatc-Borcz

**Affiliations:** Department of Organic Chemistry, Faculty of Chemical Technology and Engineering, Bydgoszcz University of Science and Technology, Seminaryjna 3, 85-326 Bydgoszcz, Poland; nina@pbs.edu.pl

**Keywords:** benzothiazole derivatives, oxime esters, spectroscopic properties, electrochemical properties, photoinitiators

## Abstract

The paper focuses on the synthesis and characterization of the spectroscopic and electrochemical properties of novel oxime esters. Six benzothiazole-based compounds were synthesized using a simple three-step procedure. The chemical structure of novel oxime esters was confirmed by Nuclear Magnetic Resonance spectroscopy (^1^H and ^13^C NMR), as well as FT-IR spectroscopy and elemental analysis. The melting point of these compounds was also determined. The spectroscopic properties were studied in 10 solvents with different polarity. The fluorescence quantum yield was determined using Coumarin I as a reference. Additionally, the E_0→0_ transition energy was determined. The electrochemical properties were determined using cyclic voltammetry. To justify their use as potential photoinitiators, preliminary studies were conducted to assess their utility in initiating light-induced polymerization. Based on the results, the proposed oxime esters are potential Type I photoinitiators for free radical polymerization.

## 1. Introduction

**Figure 1:**
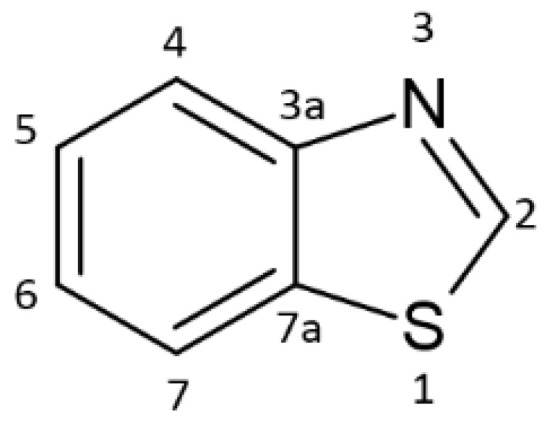
Structure of benzothiazole. Based on [2].

Benzothiazole derivatives constitute an important class of heterocyclic compounds that have attracted considerable attention due to their versatile structural modification possibilities and broad range of applications [1,2]. Substitution at the 2-position of the thiazole ring and at the 5- and 6-positions of the benzene ring enables fine tuning of their physicochemical and photophysical properties, which makes benzothiazoles attractive scaffolds for functional materials. The chemical structure of benzothiazole is presented in Figure 1 [2].

**Figure 2:**
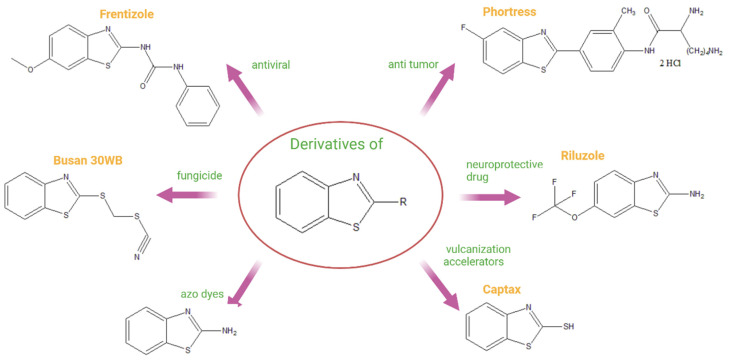
Examples of commercially available 2-substituted benzothiazole derivatives and their applications. Based on [3,4,5,6,7,8,9]. Created in BioRender. Dzwonkowska-Zarzycka, M. (2025) https://app.biorender.com/illustrations/67b837c76f44d1b94e6c635c (accessed on 25 January 2026).

Benzothiazole-based compounds have found applications in medicinal chemistry, dye synthesis, rubber vulcanization, agrochemicals, and polymer science [3,4,5,6,7,8,9]. Their wide range of applications may also be explained by biological activities, e.g., antitumor, anti-cancer, antimicrobial, antidiabetic, and antiviral properties [10]. Some examples of commercially available benzothiazole derivatives and their applications are presented in Figure 2.

Recently, several papers have been published focusing on 2-arylbenzothiazoles and justifying their use in an extensive range of photocatalytic processes [11]. There are also studies of their use as ligands in transition-metal complexes [12,13] and as chemical sensors [14,15]. Additionally, benzothiazoles are also valuable for polymer chemistry, e.g., as sensitizers/initiators in photopolymerization processes [16,17,18].

For example, three-component systems comprising a benzothiazole-based photosensitizer/*sec*-butyltriphenylborate salt/*N*-methoxy-4-phenylpyridinium salt, and optionally a diphenyliodonium salt or a 1,3,5-triazine derivative can be used as effective photoinitiators for the polymerization of acrylate monomers [19]. Another paper described two-component systems comprising a sensitizer based on a squaraine dye bearing a benzothiazole moiety, in combination with onium salts as a co-initiator [20]. Kundu and others [21] presented an interesting paper describing novel benzothiazole-based photoinitiators active in the NIR range. High quantum yields (about 0.5) and the ability to carry out the reaction in the presence of O_2_ were the main advantages of these photoinitiators.

Future work is focused on the design, preparation, and characterization of new radical photoinitiators based on the benzothiazole moiety, e.g., oxime esters.

Among various classes of photoinitiators, oxime esters have emerged as highly efficient Type I radical photoinitiators due to their easy route of synthesis and their high photoinitiating ability due to high yields of active radicals’ formation [22,23,24]. Recent research has shown that the introduction of different chromophoric groups into the oxime ester structure, such as triphenylamine, coumarin, or carbazole moieties, allows modulation of their absorption characteristics and photoinitiating performance [25,26,27]. These structural modifications enable extension of light absorption toward longer wavelengths and improvement of photopolymerization efficiency.

The aim of this paper has been the synthesis and study of the spectroscopic and electrochemical properties, as well as the photoinitiating ability, of new 2-substituted benzothiazole-based oxime esters. The selected group of oxime esters includes compounds with aliphatic and aromatic substituents. The selection of these derivatives allows analysis of the influence of minor spherical and electronic differences on spectroscopic properties, photolysis, and the kinetics of photopolymerization. Additionally, it enables comparable reactivity while limiting interfering factors, such as the volume effect associated with longer chains. This selection of oxime esters was also aimed at the subsequent optimization of the photopolymerization process, i.e., the selection of an appropriate photoinitiator for a specific monomer. The spectroscopic properties in 10 solvents with different polarity were studied. Spectroscopic analysis (^1^H and ^13^C NMR and FT-IR) confirmed the desired structures of the synthesized oxime esters. Based on the obtained results, the characteristic parameters such as: maximum of absorption (λ_max_), molar extinction coefficients (ε_max_ and ε_365 nm_), transition energy from the ground state to the excited state (E_0→0_), Stokes shift, fluorescence quantum yields, the rate constants of photodegradation, as well as the redox potentials (E_ox_ and E_red_) were determined. The kinetic study of the polymerization process confirmed that benzothiazole-based oxime esters can serve as highly efficient Type I initiators.

## 2. Materials and Methods

### 2.1. Materials

All substances, such as: solvents and chemical reagents were purchased from: Sigma-Aldrich (St. Louis, MO, USA) (diethyl ether, anhydrous acetone, propionyl chloride, acetonitrile, tetrabutylammonium perchlorate), POCH Poland (Gliwice, Poland) (sodium metabisulfite, acetone, methanol), Fisher Scientific (Hampton, NH, USA) (hydroxylammonium chloride), Chempur (Piekary Śląskie, Poland) (magnesium sulfate, tetrahydrofurane, dimethyl sulfoxide, ethyl acetate, chloroform), Thermoscientific (Waltham, MA, USA) (2-aminothiophenol, benzoyl chloride, *N*,*N*-dimethylformamide, dichloromethane), AmBeed (Buffalo Grove, IL, USA) (terephthalaldehyde, *p*-toluoyl chloride), Acros Organics (Geel, Belgium) (sodium carbonate), Alfa Aesar (Karlsruhe, Germany) (triethylamine) and Fluka Analytical (Morris Plains, NJ, USA) (acetyl chloride, dodecanoyl chloride) and used without further purification. The purity of the substrates used for synthesis was ≥98%. The solvents used in spectroscopic studies were found to be spectrally pure (≥99%).

### 2.2. Synthesis

**Figure 3:**
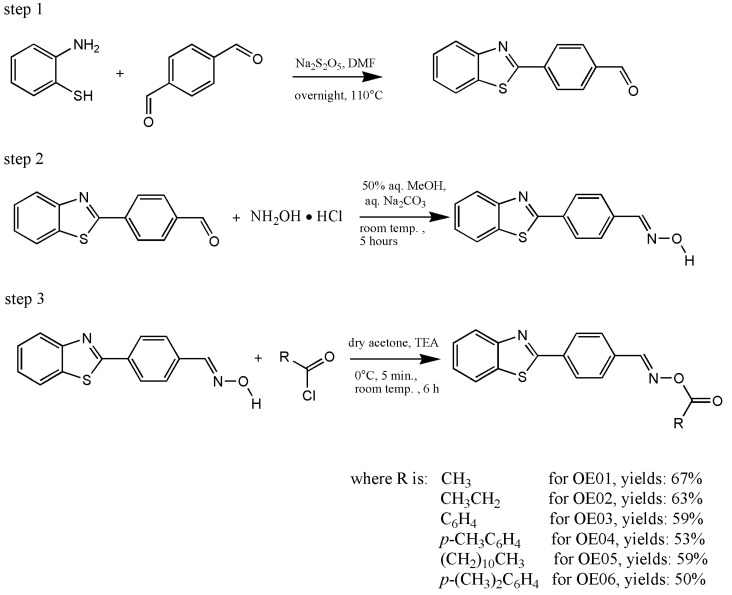
General scheme of synthesis of oxime esters. Based on [28,29,30].

All compounds were synthesized based on procedures described in the literature. The synthesis of substrates for the preparation of oxime esters, i.e., aldehyde and oxime, is described in the published papers [28,29]. The method for the synthesis of oxime esters was described by Karakurt et al. [30]. The synthetic route is shown in Figure 3.

### 2.3. Confirmation of the Chemical Structure of Synthesized Compounds

The ^1^H NMR studies were performed for the intermediate products (aldehyde and oxime). Moreover, the chemical structure of the final compounds (oxime esters) was confirmed by ^1^H and ^13^C NMR and FT-IR (ATR) spectroscopy. The ^1^H and ^13^C NMR spectra are presented in Supporting Information (Figures S1–S14). The FT-IR spectra of oxime esters are also included in Supporting Information (Figures S15–S20). The basic parameters characterizing the oxime esters synthesized are summarized below.

#### 2.3.1. [[4-(1,3-Benzothiazol-2-yl)phenyl]methyleneamino]acetate (OE01)


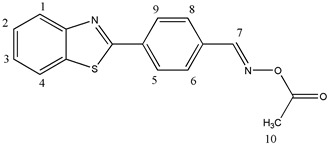


Pale yellow powder—67% yield

^1^H NMR (400 MHz, DMSO-d6), δ (ppm): 8.80 (s, 1H, H-7), 8.25–8.23 (d, 2H, H-6, H-8, *J* ≈ 8 Hz), 8.21–8.19 (d, 1H, H-1, *J* ≈ 8 Hz), 8.12–8.10 (d, 1H, H-4, *J* ≈ 8 Hz), 7.97–7.95 (d, 2H, H-5, H-9, *J* ≈ 8 Hz), 7.61–7.57 (t, 1H, H-3), 7.53–7.49 (t, 1H, H-2), 2.23 (s, 3H, H-10)

^13^C NMR, δ (ppm): 168.67, 166.72, 156.20, 154.01, 135.82, 135.16, 133.24, 129.51, 128.25, 127.37, 126.41, 123.60, 122.99, 19.85

FT-IR (ATR), *ν* (cm^−1^): 3052.74 (aromatic C-H), 1752.14 (C=O), 1605.66 (C=N)

Molecular weight—296.34 g/mol

Melting point—129.3 °C

C_16_H_12_N_2_O_2_S, Calcd.: C, 64.85; H, 4.08; N, 9.45. Found: C, 65.10; H, 4.13; N, 9.30.

#### 2.3.2. [[4-(1,3-Benzothiazol-2-yl)phenyl]methyleneamino]propanate (OE02)


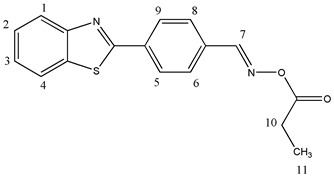


Pale yellow powder—63% yield

^1^H NMR (400 MHz, DMSO-d6), δ (ppm): 8.80 (s, 1H, H-7), 8.25–8.23 (d, 2H, H-6, H-8, *J* ≈ 8 Hz), 8.21–8.19 (d, 1H, H-1, *J* ≈ 8 Hz), 8.12–8.10 (d, 1H, H-4, *J* ≈ 8 Hz), 7.97–7.95 (d, 2H, H-5, H-9, *J* ≈ 8 Hz), 7.61–7.57 (t, 1H, H-3), 7.53–7.49 (t, 1H, H-2), 2.58–2.54 (t, 2H, H-10), 1.12–1.15 (t, 3H, H-11)

^13^C NMR, δ (ppm): 171.91, 166.72, 156.28, 154.01, 135.80, 135.16, 133.27, 129.49, 128.25, 127.36, 126.40, 123.59, 122.98, 25.87, 9.27

FT-IR (ATR), *ν* (cm^−1^): 3020.23 (aromatic C-H), 1761.41 (C=O), 1607.19 (C=N)

Molecular weight—310.37 g/mol

Melting point—148.5 °C

C_17_H_14_N_2_O_2_S, Calcd.: C, 65.79; H, 4.55; N, 9.03. Found: C, 66.17; H, 4.79; N, 8.70.

#### 2.3.3. [[4-(1,3-Benzothiazol-2-yl)phenyl]methyleneamino]benzoate (OE03)


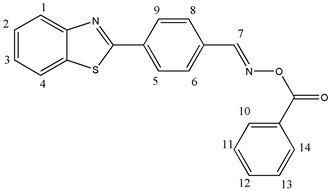


Pale yellow powder—59% yield

^1^H NMR (400 MHz, DMSO-d6), δ (ppm): 9.06 (s, 1H, H-7), 8.31–8.27 (m, 2H, H-6, H-8), 8.22–8.20 (d, 1H, H-1, *J* ≈ 8 Hz), 8.14–8.10 (m, 3H, H-4, H-10, H-14), 8.05–8.02 (d, 2H, H-5, H-9, *J* ≈ 12 Hz), 7.78–7.74 (t, 1H, H-3), 7.65–7.58 (m, 3H, H-11, H-12, H-13), 7.54–7.52 (t, 1H, H-2)

^13^C NMR, δ (ppm): 166.71, 163.58, 157.78, 154.02, 135.99, 135.18, 134.44, 133.08, 129.81, 129.67, 129.53, 128.61, 128.33, 127.38, 126.43, 123.62, 123.00

FT-IR (ATR), *ν* (cm^−1^): 3029.84 (aromatic C-H), 1733.66 (C=O), 1614.25 (C=N)

Molecular weight—358.41 g/mol

Melting point—163.3 °C

C_21_H_14_N_2_O_2_S, Calcd.: C, 70.37; H, 3.94; N, 7.82. Found: C, 70.60; H, 4.23; N, 7.50.

#### 2.3.4. [[4-(1,3-Benzothiazol-2-yl)phenyl]methyleneamino]-4-methylbenzoate (OE04)


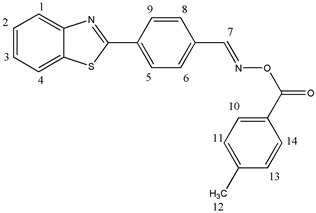


Pale yellow powder—53% yield

^1^H NMR (400 MHz, DMSO-d6), δ (ppm): 9.03 (s, 1H, H-7), 8.31–8.26 (m, 2H, H-6, H-8), 8.22–8.20 (d, 1H, H-1, *J* ≈ 8 Hz), 8.14–8.11 (m, 1H, H-4), 8.03–7.99 (m, 4H, H-5, H-9, H-10, H-14), 7.62–7.58 (t, 1H, H-3), 7.54–7.50 (t, 1H, H-2), 7.46–7.42 (t, 2H, H-11, H-13), 2.43 (s, 3H, H-12)

^13^C NMR, δ (ppm): 166.72, 163.55, 157.55, 154.02, 144.94, 135.95, 135.18, 130.09, 129.86, 129.63, 128.61, 128.32, 127.38, 126.42, 125.66, 123.61, 123.00, 21.72

FT-IR (ATR), *ν* (cm^−1^): 3029.15 (aromatic C-H), 1731.80 (C=O), 1611.52 (C=N)

Molecular weight—372.44 g/mol

Melting point—162.1 °C

C_22_H_16_N_2_O_2_S, Calcd.: C, 70.95; H, 4.33; N, 7.52. Found: C, 70.78; H, 4.70; N, 7.83.

#### 2.3.5. [[4-(1,3-Benzothiazol-2-yl)phenyl]methyleneamino]dodecanoate (OE05)


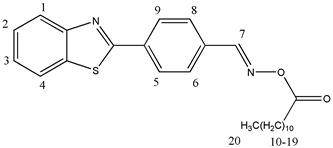


Pale yellow powder—59% yield

^1^H NMR (400 MHz, DMSO-d6), δ (ppm): 8.79 (s, 1H, H-7), 8.32 (s, 1H, H-6), 8.25–8.19 (m, 3H, H-5, H-9, H-8), 8.15–8.10 (t,1H, H-1), 7.97–7.95 (d, 1H, H-4, *J* ≈ 8 Hz), 7.62–7.57 (m, 1H, H-3), 7.54–7.49 (m, 1H, H-2), 1.64–1.61 (m, 2H, H-10), 1.25 (s, 18H, H-11–H-19), 0.87–0.83 (t, 3H, H-20)

^13^C NMR, δ (ppm): 171.05, 166.73, 156.35, 154.01, 135.16, 129.50, 128.61, 128.25, 127.37, 126.41, 123.60, 123.00, 32.39, 31.75, 29.44, 29.31, 29.16, 29.10, 28.86, 24.73, 22.55, 14.41

FT-IR (ATR), *ν* (cm^−1^): 3034.10 (aromatic C-H), 1751.99 (C=O), 1606.18 (C=N)

Molecular weight—436.61 g/mol

Melting point—150 °C

C_26_H_32_N_2_O_2_S, Calcd.: C, 71.52; H, 7.39; N, 6.42. Found: C, 71.91; H, 7.48; N, 6.28.

#### 2.3.6. [[4-(1,3-Benzothiazol-2-yl)phenyl]methyleneamino]-4-(N,N-dimethylamino)benzoate (OE06)


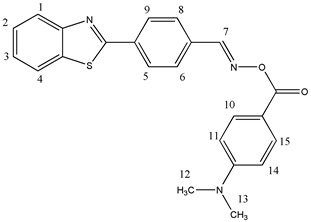


Pale yellow powder—50% yield

^1^H NMR (400 MHz, DMSO-d6), δ (ppm): 8.25 (s, 1H, H-7), 8.18–8.16 (d, 1H, H-5, *J* ≈ 8 Hz), 8.15–8.13 (d, 2H, H-6, H-8, *J* ≈ 8 Hz), 8.09–8.07 (d, 1H, H-9, *J* ≈ 8 Hz), 7.86–7.83 (d, 1H, H-1, *J* ≈ 12 Hz), 7.81–7.78 (d, 2H, H-10, H-15, *J* ≈ 12 Hz), 7.76–7.74 (d, 1H, H-4, *J* ≈ 8 Hz), 7.59–7.55 (m, 1H, H-3), 7.51–7.47 (m, 1H, H-2), 6.80–6.78 (d, 1H, H-11, *J* ≈ 8 Hz), 6.72–6.69 (d, 1H, H-14, *J* ≈ 12 Hz), 2.99 (s, 6H, H-12, H-13)

^13^C NMR, δ (ppm): 167.99, 163.51, 154.66, 154.05, 153.56, 134.99, 133.74, 132.54, 131.37, 128.05, 127.71, 127.24, 126.16, 123.41, 122.88, 117.39, 111.22, 40.10, 40.07

FT-IR (ATR), *ν* (cm^−1^): 3046.76 (aromatic C-H), 1747.58 (C=O), 1595.16 (C=N)

Molecular weight—401.48 g/mol

Melting point—194.6 °C

C_23_H_19_N_3_O_2_S, Calcd.: C, 68.81; H, 4.77; N, 10.47. Found: C, 68.54; H, 4.87; N, 10.15.

### 2.4. Methods

#### 2.4.1. NMR Measurements

The ^1^H and ^13^C NMR spectra were recorded using an Ascend III spectrometer operating at 400 MHz (Bruker, Billerica, MA, USA). Depending on the solubility of synthesized compounds, deuterated chloroform (CDCl_3_–d) or dimethyl sulfoxide (DMSO–d6) was used as a solvent. Tetramethylsilane (TMS) served as the internal standard. The chemical shifts (*δ*) are reported in ppm, and coupling constants (*J*) are expressed in Hz.

#### 2.4.2. FT-IR (ATR)

Infrared spectra were measured using a Bruker Alpha compact FT-IR spectrometer equipped with a diamond ATR attachment (Bruker). The IR spectra were recorded in the range of 4000–360 cm^−1^.

#### 2.4.3. Elemental Analysis

The elemental analysis was conducted with a Vario MACRO 11.45–0000, Elemental Analyzer System GmbH (Langenselbold, Germany), operating with the VARIOEL software (version 5.14.4.22).

#### 2.4.4. Melting Point Measurements

The melting points were measured on the Melting Point M-565 Apparatus (Buchi, Tokyo, Japan). The heating rate was 5 °C/min.

#### 2.4.5. Spectroscopic Measurements

The absorption spectra were recorded at room temperature in the range from 200 to 800 nm in a quartz cuvette (1 cm) using UV–Vis Cary 60 Spectrophotometer (Agilent Technologies, Santa Clara, CA, USA). The absorption spectra were registered for compounds dissolved in various solvents. The molar extinction coefficients (ε_max_ and ε_365 nm_) were calculated using the Beer-Lambert law.

The emission spectra were recorded at room temperature in a quartz cuvette using a Hitachi F-7000 Spectrofluorometer (Hitachi, Tokyo, Japan) with an excitation wavelength of 320 nm.

The fluorescence quantum yields of all oxime esters were determined using diluted solutions of these compounds, with an absorbance at 366 nm of 0.2. Coumarin I in ethanol as a solvent was used as the reference. The values of quantum yield were calculated using the following formula—Equation (1) [31]:

(1) $$\Phi_{dye}=\Phi_{ref}*\frac{I_{dye}*A_{ref}{{*n}_{dye}}^{2}}{I_{ref*}A_{dye}*{n_{ref}}^{2}}$$

where Φ*_ref_*—fluorescence quantum yield of reference, I*_dye_*, I*_ref_*—integrated fluorescence intensity of dye and reference, A*_dye_*, A*_ref_*—absorbance of dye and reference at the excitation wavelength, n*_dye_*, n*_ref_*—refractive index of solvents used for dye and reference, respectively. The value of Φ*_ref_* for Coumarin I used for calculations was 0.64 [32]. 

#### 2.4.6. Electrochemical Measurements

Cyclic voltammetry (CV) studies were performed using an ER466 Integrated Potentiostat System (eDAQ, Warsaw, Poland) in a three-electrode configuration. The working electrode was a 1 mm platinum disk, whereas a platinum wire and a silver chloride electrode (Ag/AgCl) were used as auxiliary and reference electrodes, respectively. The computer–controlled potentiostat equipment with EChem software (EAlab 2.1) enabled processing of recorded current–potential curves. The role of the electrolyte took the solution of 0.1 M tetrabutylammonium perchlorate in acetonitrile. All solutions were deoxygenated with nitrogen before each measurement.

#### 2.4.7. Steady-State Photolysis

Photolysis experiments were performed in acetonitrile as a solvent. The concentration of each oxime ester was 2.98 × 10^−5^ M. The absorption spectra were recorded at room temperature over the range of 200–600 nm in a 1 cm quartz cuvette using an Agilent Technologies Cary 60 UV–Vis spectrophotometer (Agilent Technologies, USA). The solution was exposed to external light—an LED Lamp with a wavelength of 365 nm and light intensity of 50 mW/cm^2^. The measurements were carried out over selected time intervals (0, 1, 2, 5, 10, 15, 20, 25, and 30 min). The kinetics of photodegradation of oxime esters were determined using the following Equation (2) [33]:

(2) $$\;\ln\left(\frac{10^{A}-1}{10^{A_{0}}-1}\right)\;=-k_{bl}\;\times \;t$$

where *A*_0_ and *A* are the absorbance of the dye at 365 nm at time zero and *t*, respectively. The *k_bl_* is the photobleaching constant.

#### 2.4.8. Photopolymerization Experiments

The photopolymerization kinetics were studied using real-time Fourier Transform Infrared Spectroscopy (FTIR) with a NICOLET iS10 FTIR spectrometer (Thermo Scientific^TM^, Thermo Fisher Scientific, Waltham, MA, USA) equipped with a horizontal attachment. All measurements were conducted at 25 °C using the same light source as for steady-state photolysis experiments, i.e., an LED at 365 nm (M365L2, Thorlabs Inc., Newton, NJ, USA). The diode operating parameters, i.e., current and the corresponding light intensity on the sample surface, were set accordingly. Detailed experimental conditions for each measurement series are provided in the figure captions. The sample was irradiated for 10 s after the process began. The distance between the irradiation source and the polymerizable formulation was 2.1 cm. To reduce the oxygen inhibition, thin photocurable compositions (25 µm thick) were sandwiched between two propylene films deposited on a magnetic holder in an FTIR apparatus and then irradiated.

The formulations were composed of propylene carbonate (PC), monomer (TMPTA) and an appropriate photoinitiator. The use of propylene carbonate was necessary for the preparation of the samples because of the poor solubility of oxime esters in the monomer composition (solvent-monomer volume ratio was 1:9).

The OMNIC software (OMNIC 9.2) enabled continuous monitoring of photopolymerization progress by observing the intensity of the [ʋ(C=C)] band characteristics for the corresponding monomers (at about 1636 cm^−1^). The final conversion of functional groups was calculated using Equation (3) [34]:

(3) $$\%\;C_{FTIR}=(1-\frac{A_{after}}{A_{before}})\;\times \;100\%$$

where % *C_FTIR_* is the conversion of a given monomer/functional group of a monomer, *A_after_* is an area of the absorbance peak characteristic of a given monomer at the end of the photopolymerization process, and *A_before_* is an area of the absorbance peak characteristic of a given monomer at the beginning of the photopolymerization process.

The rate of polymerization (*R_p_*) was determined from the slope of the conversion vs. time plot, at the initial stage of polymerization, using the following Equation (4) [35]:

(4) $$R_{p}=\frac{dC_{FTIR}}{dt}\;\times \;100\%$$

The value of *R_p_* is a measure of the maximum rate of photopolymerization and indicates the photoinitiator’s efficiency.

## 3. Results and Discussion

### 3.1. Synthesis

Nowadays, the scientific community is paying close attention to the search for highly effective synthetic methods for novel compounds. The synthesis of oxime esters was carried out in three steps. The general synthetic procedure is shown in Figure 3.

The first step was the preparation of benzothiazole-based aldehyde. Generally, three basic methods for the synthesis of 2-substituted benzothiazole aldehyde can be found in the literature: (1) condensation reaction of 2-aminothiophenol with carbonyl- or cyano-group compounds [36], (2) reaction between *ortho*-halogenated aniline with aldehydes, sulfur, thiol or Lawesson’s agent [37,38], (3) intramolecular cyclization of *ortho*-halogenated analogs [39]. These methods can have many drawbacks, including low efficiency, the use of toxic solvents, poor selectivity, or high temperatures. The alternative procedure was proposed by Ye and co-workers [40]. This method is suitable for aromatic, aliphatic, and heteroatomic aldehydes. It proceeds under milder conditions: a visible-light source (a 12 W blue LED), in air, and at a reduced temperature below the boiling point of ethyl acetate. Another alternative method involves condensing 2-aminothiophenol with aryl aldehydes. The method was developed by Maphupha and co-workers [41] and assumes conducting the reaction at room temperature, using laccase as a catalyst and achieving high efficiency (56–88%). The method selected for our studies was also characterized by high efficiency (about 70–90%) and simplicity. The reactants used were easy to obtain, and the reaction products did not require complicated purification procedures. Although organic solvents were used, their quantity was not significant.

Among the methods of oxime synthesis, several procedures differ in temperature, reaction time, and environment. A common feature is the use of hydroxylamine hydrochloride (NH_2_OH⸱HCl) as a carrier of the N-OH group.

The procedure proposed in 2021 includes the use of anhydrous sodium acetate, ethanol and a nitrogen atmosphere overnight at reflux temperature. The reaction yield was 92%, confirming the method’s efficiency for the synthesis of coumarin-based oximes [26]. Song and co-workers modified this method. The high yields of reaction (87%) were obtained [42]. On the other hand, Damljanović and co-workers used a solvent-free method. The advantages of this method include a short reaction time (30–120 min), simplicity (ground in a mortar), high efficiency (72–100%), and mild reaction conditions (no solvent required, at room temperature). The method proved helpful in the synthesis of oximes from corresponding aliphatic or alicyclic ketones in the presence of NaOH [43]. The oxime synthesis method chosen was the result of testing all the aforementioned procedures. Low reaction yields or decomposition of products (determined by ^1^H NMR analysis) led to the selection of a solvent-assisted synthesis method in 50% aqueous methanol, using sodium carbonate. Structural analysis confirmed the efficiency of the chosen method and the compound’s qualification of the compound for further reactions.

Among the strategies for synthesizing oxime esters, three main strategies are commonly used and reported in the literature. Reactions between oximes and carboxylic acids, aldehydes or esters give the oxime esters [44]. The method used was based on the application of triethylamine (which deprotonates the oxime) and stirring the reaction mixture for 5 min at 0° C, then for 6 h at room temperature, followed by the addition of an acid chloride (known for its high reactivity). Thirty minutes after adding the chloride, the product was obtained, and the solvent was removed by evaporation; the residue was then extracted with water and cold ether [30].

Subsequent analyses performed confirmed the effectiveness of the methods used and characterized the compounds obtained.

All synthesized compounds are yellow powders. Due to differences in chemical structure, oxime esters exhibited a range of melting points. The melting points of oxime esters increase in the following order: OE01 < OE02 < OE05 < OE04 < OE03 < OE06.

The NMR and FTIR spectra confirmed chemical structures of all synthesized compounds. The ^1^H NMR spectra showed the presence of peaks characteristic of the given protons. All of the signals in the region between 7.4 ppm and 7.7 ppm confirm the presence of protons (in the form of triplets) of the benzothiazole ring. For aldehyde (A2B), the characteristic peak was observed at 9–11 ppm, confirming the presence of the -CHO group (δ = 10.03 ppm). The -NOH group was observed in the region of approx. 11 ppm (δ = 11.55 ppm). Oxime esters with an attached aliphatic group should be characterized by the presence of peaks in the range from 0 to 6 ppm. The analysis of ^1^H NMR spectra confirmed the presence of these groups in the esters OE01, OE02, OE04, OE05, and OE06. The introduction of an additional phenyl ring led to the appearance of signals between 7 and 9 ppm for OE03, OE04, and OE06, respectively. Additional confirmation of the effectiveness of the syntheses of the obtained compounds was provided by ^13^C Nuclear Magnetic Resonance analysis. The presence of characteristic peaks in the ^13^C NMR spectra corresponding to -C=N and -C=O groups confirmed the formation of the oxime ester. The signals located at approximately 154 ppm and between 163 and 166 ppm confirm the presence of -C=N and -C=O bonds, respectively.

FT-IR (ATR) spectroscopy also revealed bands characteristic of the given groups. The C=H aromatic bond was found at about 3030 cm^−1^. The C=O and C=N bond peaks appeared at ca. 1731–1761 cm^−1^ and 1595–1614 cm^−1^, respectively.

Based on literature analysis [45] and ^1^H NMR spectra, the configuration of the resulting oxime esters was determined. The configuration type was assigned by analyzing the proton found attached to the carbon double-bonded to nitrogen. In all oxime esters, this signal was in the range of 8.25 ppm (OE06) and between 8.8 ppm and 9.6 ppm. Such a high signal intensity may suggest that the obtained esters have a *Z* configuration. In these types of compounds, the *E* configuration proton signal occurs in the lower ppm range. Additionally, since this signal is always a singlet, it confirms that only one configuration was obtained. If it were a mixture, the signal would be a doublet.

### 3.2. Spectroscopic Properties

The spectroscopic properties of oxime esters were studied in 10 solvents of varying polarity. The solvents used, arranged according to increasing values of dielectric constants, are listed below [46,47]:

Diethyl ether (Et_2_O—4.33) < chloroform (CHCl_3_—4.81) < ethyl acetate (AcOEt—6.02) < tetrahydrofurane (THF—7.4) < dichloromethane (DCM—8.93) < acetone (ACE—20.7) < methanol (MeOH—32.7) < *N*,*N*—dimethylformamide (DMF—36.7) < acetonitrile (MeCN—37.5) < dimethyl sulfoxide (DMSO—46.7). The spectroscopic properties of oxime esters studied are presented in Table 1.

**Figure 4:**
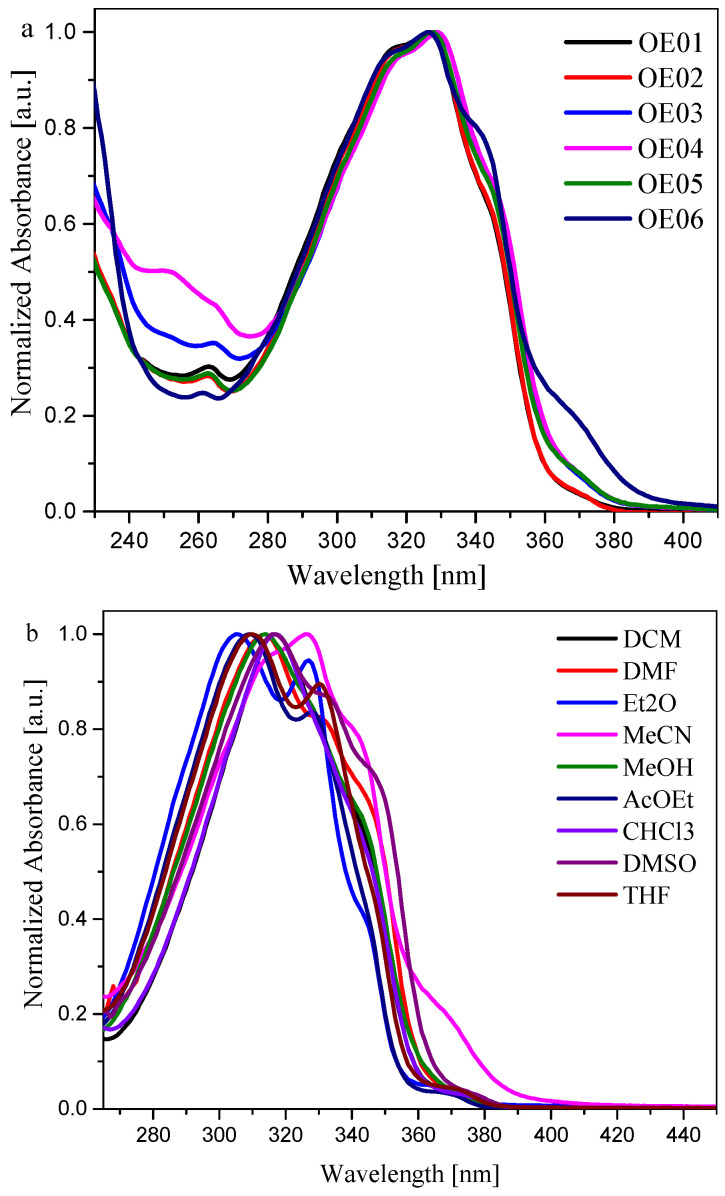
(**a**) Normalized absorption spectra of all 2-substituted oxime esters in acetonitrile (MeCN). (**b**) Normalized absorption spectra of OE06 in different solvents.

All oxime esters studied absorb between 270 nm and 390 nm, with a broad band centered at about 310–330 nm (Figure 4a,b). This band is attributed to the π→π* transition. It should be noted that the λ_max_ absorption values are similar for OE01, OE02, OE03, OE04, and OE05. On the other hand, the maximum of absorption for OE06 appeared at a shorter wavelength, about 315 nm, which may be due to the presence of the -N(CH_3_)_2_ group attached to the benzene ring in the R substituent.

The values of λ_max_ absorption do not depend on the polarity of the solvent. The solvatochromic shift in the absorption band caused by the increase in polarity of the solvent is slight and amounts to 5 nm (for OE02) and 9 nm (for OE03 and OE05). The type of substituent in the oxime ester moiety does not cause a significant shift in the maximum absorption and the shape of the absorption band. It was found that solvent polarity does not significantly affect the spectroscopic properties of the synthesized dyes.

The value of the molar extinction coefficient (ε_max_) determines the ability of the chromophore for light absorption [48]. The molar extinction coefficient at λ_max_ (ε_max_) differs for each oxime ester and depends on the solvent used. The value of ε_max_ for OE01 was in the range between 15,039 M^−1^ · cm^−1^ (ACE) and 25,006 M^−1^ · cm^−1^ in MeCN. This oxime ester possesses the highest value of the molar extinction coefficient. On the other hand, the molar extinction coefficient for OE06 ranges from 2749 M^−1^ · cm^−1^ (MeCN) to 7498 M^−1^ · cm^−1^ (ACE). For other compounds, this parameter was approximately 10 times lower than for OE01. Given the possibility of using these compounds as photoinitiators, a UV light lamp should be chosen as the light source. Due to absorption in the 270 nm–400 nm range, a photosensitive composition containing oxime esters can be irradiated with a UV/LED, for example, a 365 nm LED.

Taking into account the effect of polarity of the solvent on spectroscopic properties of oxime esters, it can be said that the solvent has an insignificant effect on the position of the absorption band (see Table 1). It should be noted that the absorption bands of oxime esters in DMSO and CHCl_3_ shift towards longer wavelengths.

Although the effect of solvent polarity on the position of the absorption bands is negligible, the changes in the maximum absorption values are discernible. For example, the absorption maximum is shifted toward shorter wavelengths (λ_max_ = 305 nm). In contrast, in a more polar solvent (i.e., MeCN), the value of λ_max_ is shifted toward longer wavelengths (λ_max_ = 326 nm).

In order to determine the influence of the solvent polarity on the spectral properties of novel oxime esters, the correlations between the solvent polarity parameter and the position of absorption bands were designated. The scale that reflects this relationship is the Dimroth–Reichardt scale. This scale is based directly on the spectrophotometric determination of λ_max_ and is defined as the molar transition energies (in kcal mol^−1^) of the standard betaine dye. This parameter can be calculated using the formula (Equation (5)) [49]:

(5) $$E_{T}\left(30\right)=28591/\lambda_{max}\;(\mathrm{nm})$$

Another scale that tests the polarity of compounds is the Kamlet-Taft scale. The physical properties of the solvents used for calculations are summarized in Table 2.

The R^2^ values calculated by the aforementioned methods are summarized in Table 3. The R^2^ values are low enough that no correlation can be found between the polarity parameter and the spectroscopic properties. These calculations confirmed the conclusions mentioned above (see Table 1).

Fluorescence properties of molecules are among the fundamental information needed to understand their photophysical and photochemical properties, particularly their singlet states [50]. The 2-substituted benzothiazole-based oxime esters emit light from 335 nm to 600 nm. All compounds showed a single fluorescence band with a maximum between 387 nm and 408 nm. Based on the Kamlet-Taft scale, the effect of solvent polarity on the maximum of the fluorescence band was determined. The summary of the R^2^ parameter calculated using both scales is presented in Table 4.

**Figure 5:**
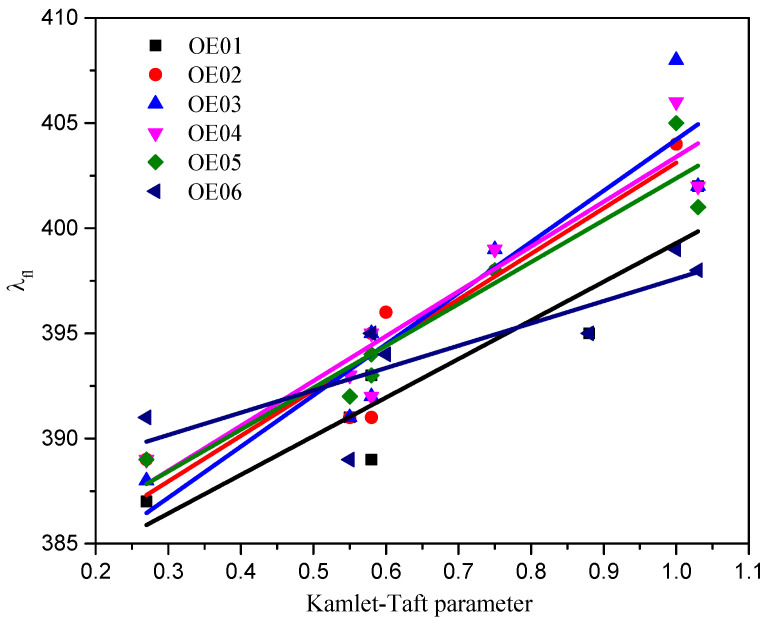
The correlation between λ_fl_ and Kamlet-Taft solvent parameter for oxime esters.

The correlation between λ_fl_ and Kamlet–Taft parameter is shown in Figure 5. It should be pointed out that the type of solvent has a considerable effect on the fluorescence of oxime esters. Based on the values of R^2^ (Kamlet–Taft scale), one can conclude that, with an increase in the Kamlet—Taft parameter, the λ_fl_ value shifts toward longer wavelengths.

**Figure 6:**
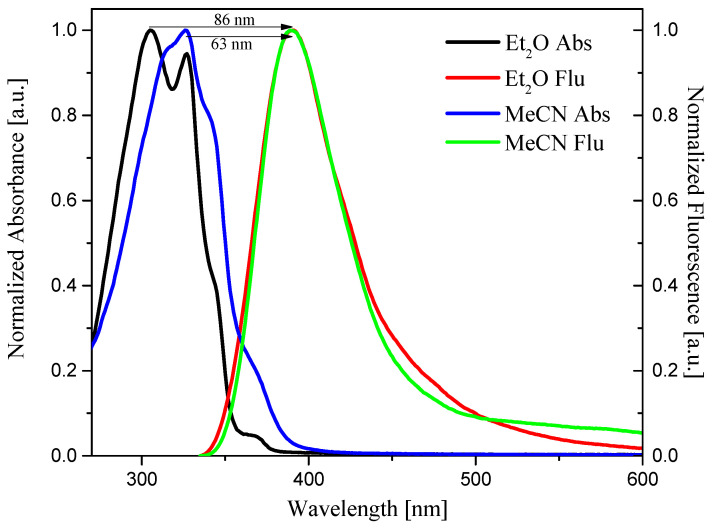
Stokes Shift of OE06 in two different solvents—Et_2_O and MeCN.

The Stokes shift values for all compounds ranged from 4596 cm^−1^ to 7211 cm^−1^ and depended on the oxime ester structure and the solvent type. However, on average, the highest values were observed for OE06. Generally, an increase in solvent polarity was found to increase the Stokes Shift, except for ester OE06. Figure 6 presents the influence of the type of solvent used on the value of the Stokes shift for OE06.

**Figure 7:**
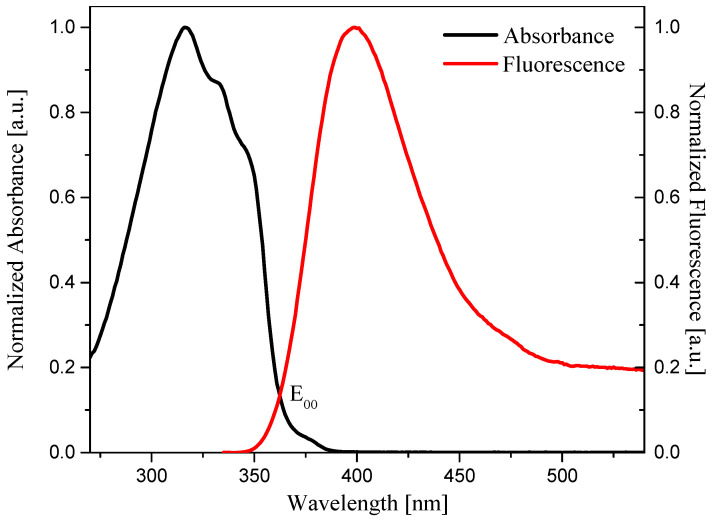
Determination of E_0→0_ for OE06 (solvent: DMSO).

Normalization of the absorption and fluorescence spectra allows determination of the transition energy from the ground state to the excited state (E_0→0_). The value of E_0→0_ was determined from the intersection point of the above-mentioned spectra. The lowest transition energy was obtained for OE06 (Figure 7) and OE05, both at 3.38 eV. For all oxime esters, the lowest E_0→0_ were observed in the most polar solvent—DMSO. The highest values of this parameter were obtained in THF for compounds OE01, OE02, OE05, and OE06, with a value of 3.50 eV.

Another parameter describing fluorescence properties is the fluorescence quantum yield (Φ_fl_), which defines the ratio of the number of photons emitted by the compound to the number of photons absorbed [50]. All values of Φ_fl_ are shown in Table 5.

The values of Φ_fl_ presented in Table 5 are low. The fluorescence quantum yields for all compounds range from 0.0507 (for OE03 in DMSO) to 0.1713 (for OE02 in THF). It can be noted that the value of Φ_fl_ affects the type of solvent used. The highest values for each oxime esters were obtained in tetrahydrofuran (THF), and the lowest in dimethyl sulfoxide (DMSO). The high polarity of DMSO can cause faster deactivation of the excited state and the transition to the ground state when the fluorescence process is omitted (e.g., due to heat emission). In high-polarity solvents, non-radiative deactivation of the excited state of oxime esters prevails over radiative deactivation.

### 3.3. Electrochemical Properties

**Figure 8:**
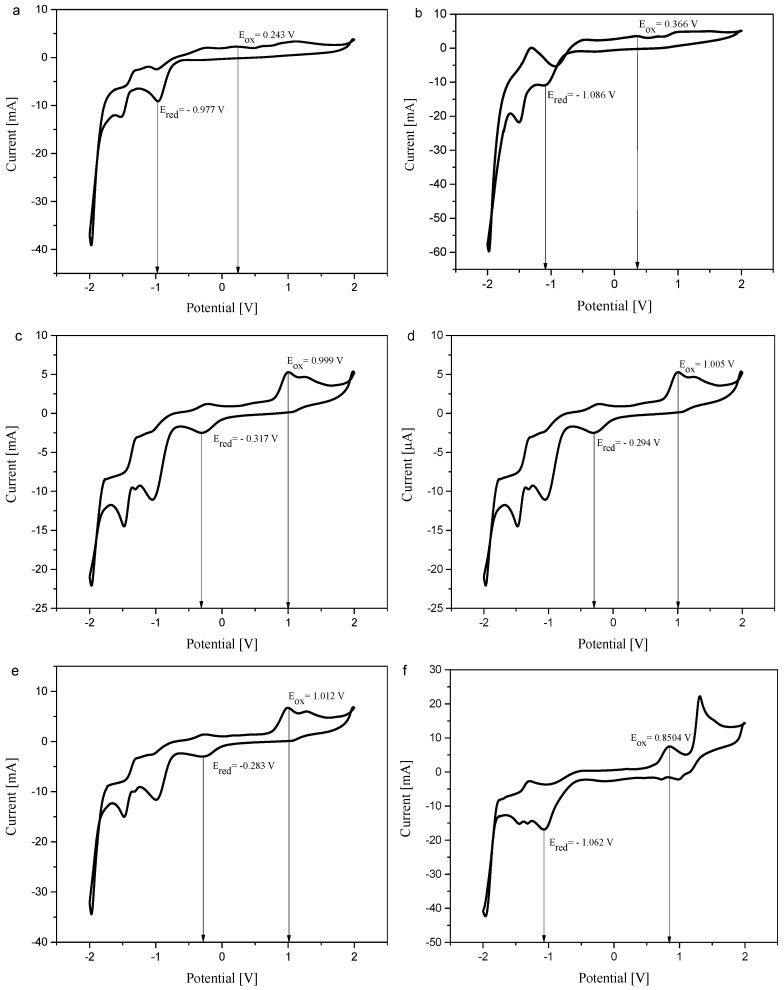
(**a**–**f**). The oxidation and reduction process determination in cyclic voltammetry of benzothiazole–based oxime esters (**a**) OE01, (**b**) OE02, (**c**) OE03, (**d**) OE04, (**e**) OE05, (**f**) OE06.

The photooxidative or photoreductive properties of oxime esters were estimated by cyclic voltammetry (CV). All results are summarized in Table 6. Figure 8a–f shows a graphical presentation of the electrochemical properties of benzothiazole-based oxime esters. The oxime esters studied undergo both oxidation and reduction processes. The oxidation potentials for all compounds range from 0.243 V to 1.012 V. The reduction potential ranged from −1.083 V to −0.283 V. In order to evaluate the possibility of interaction between oxime ester and other compounds (i.e., co-initiator) in photopolymerization experiments, the Gibbs energy should be calculated. Further, the change in free energy of the electron transfer process (ΔG_el_) will be determined based on the classical Rehm-Weller equation (Equation (6)):

(6) $$\Delta G_{el}=\;E_{ox}\;-{\;E}_{red}\;-\;E^{*}\;+\;C\;$$

where

*E_ox_*—the oxidation potential of an electron donor, *E_red_*—the reduction potential of an electron acceptor, E*—the excited state energy level (calculated from the intersection of normalized absorption and fluorescence spectra), *C*—the coulombic term for the initially formed ion pair (ignored for polar solvents) [51].

**Table 6:** Electrochemical properties of benzothiazole-based oxime esters.

Dye	Transition Energy	Redox Potentials	
	E_0→0_ ^a^ [eV]	E_ox_ [V]	E_red_ [V]
OE01	3.45	0.243	−0.977
OE02	3.45	0.366	−1.086
OE03	3.42	0.999	−0.317
OE04	3.42	1.005	−0.294
OE05	3.43	1.012	−0.283
OE06	3.44	0.850	−1.062

^a^ Measured in MeCN.

The redox potentials reported in this publication will be used to define the role of the oxime ester as a type II photoinitiator in combination with a co-initiator for the photopolymerization reaction.

### 3.4. Steady-State Photolysis

**Figure 9:**
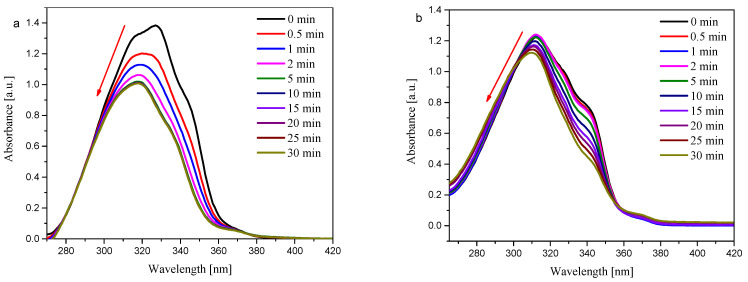
Photolysis of (**a**) OE01 and (**b**) OE06 in acetonitrile under @LED 365 nm with a light intensity (50 mW/cm^2^) irradiation.

The results of the photolysis carried out under irradiation with a wavelength of 365 nm were presented as graphs and included in Supporting Information (Figures S37–S42). Examples of two oxime esters, OE01 and OE06, are shown in Figure 9.

In all esters, irradiation at 365 nm and photochemical decomposition were observed. In addition, as the absorbance decreased during exposure, a shift in the absorption spectrum toward shorter wavelengths was noted. The type of substituent influenced the rate of photodegradation of the oxime ester moiety. From Equation (2), and the slope of the linear relationship between absorbance at the time of irradiation, the rate constants of photodecay were calculated. The results obtained are summarized in Table 7.

The highest photodegradation rate was observed for OE04, which possesses an aromatic ring with a methyl substituent. The aforementioned compound should also serve as a photoinitiator during polymerization. This type I photoinitiator should exhibit the fastest rate in the initial phase of polymerization initiation. The average rates of photodegradation were observed for oxime esters with aliphatic substituents (OE01, OE02, OE05), and for OE03 with an aromatic ring as a substituent. The most photostable compound was the oxime ester OE06.

### 3.5. Photopolymerization Experiments

**Figure 10:**
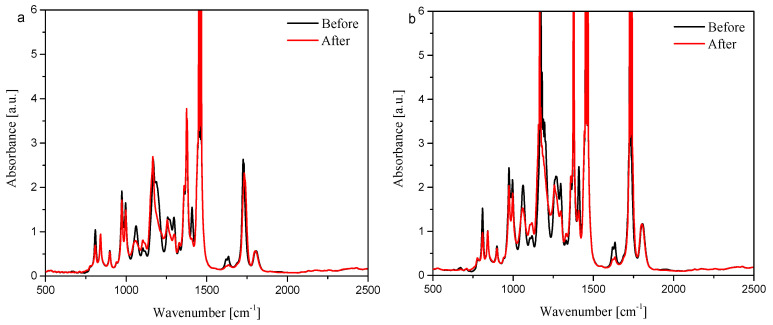
RT-FTIR spectra recorded during radical polymerization of TMPTA initiated by oxime esters, (**a**) OE02 and (**b**) OE03, as photoinitiators with a concentration of 2%, light intensity 28 mW/cm^2^ (@LED365 nm).

The RT-IR (Fourier-transform real-time infrared spectroscopy) technique is an effective method for monitoring photopolymerization. In this case, the study consisted of repeatedly scanning the IR spectrum and observing changes in the intensity at 1636 cm^−1^, characteristic of the C=C stretching vibration (TMPTA). Changes in the intensity of this band were noted, as shown in Figure 10.

Regardless of the chosen compound (aliphatic or aromatic substituent), a decrease in the intensity of the characteristic absorption band at 1636 cm^−1^ (changes before and after irradiation) was observed. Based on this, these compounds may be used as potential photoinitiators, effectively initiating light-induced polymerization of acrylates (in this case, TMPTA).

To confirm the applicability of the obtained oxime esters as potential Type I initiators, the optimization of the photopolymerization process was performed for two compounds possessing the aliphatic (OE02) and aromatic (OE03) substituents. The effect of photoinitiator concentration and chemical structure, as well as light intensity, on the photopolymerization process was studied. TMPTA was used as a monomer. The results are summarized in Table 8 and Table 9. Some correlations were observed.

**Figure 11:**
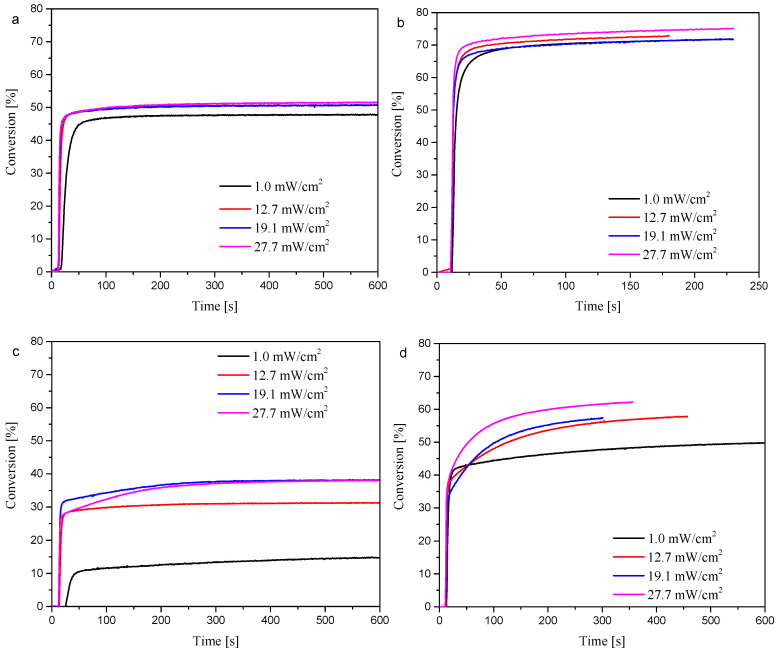
Photopolymerization profiles for TMPTA under LED 365 nm irradiation for (**a**) 0.1% wt of OE02; (**b**) 2% wt of OE02; (**c**) 0.1% wt of OE03; (**d**) 2% wt of OE03.

The maximum degree of monomer conversion was 74.5% (2% *w*/*w*, 27.7 mW/cm^2^) for OE02. Under the same experimental conditions, the photoinitiator OE03 yielded a monomer conversion of about 61%. It was found that increasing the initiator concentration increased the degree of monomer conversion for both photoinitiators containing an aliphatic substituent (OE02) and an aromatic substituent (OE03). At the same time, increasing concentration decreased the induction time. This parameter was also affected by light intensity: the higher it was, the shorter the induction time. Figure 11 shows the change in conversion per unit time for two oxime esters, OE02 and OE03, at different light intensities but equal concentrations.

The RT-FTIR technique also allows determination of the reaction rate (R_p_), thereby enabling the compound to be more accurately identified and qualified as a potential photoinitiator. The results are summarized in Table 10. In the compared concentration variants (0.1% and 2%), the oxime ester with an aliphatic substituent showed a rate of polymerization higher than the ester with an aromatic substituent. In addition, the higher the light intensity, the higher the reaction rate.

Based on these results, combining the two groups of compounds (oxime esters and benzothiazoles) yields good monomer conversion, providing an additional argument for the potential of this group.

## 4. Conclusions

In this paper, a series of six novel 2-substituted benzothiazole-based oxime esters was synthesized. The structure of novel compounds was confirmed by Nuclear Magnetic Resonance (^1^H and ^13^C NMR), as well as FT-IR spectroscopy and elemental analysis. The spectroscopic properties of these compounds were determined in ten solvents of different polarity. A linear correlation between λ_fl_ and the Kamlet-Taft parameters was observed. The benzothiazole-based compounds showed a similar absorption range. The presented results will be valuable for studies of the ability of oxime esters to act as Type I initiators in photopolymerization under 365 nm light. The determined values of oxidation and reduction potentials will define the role of the oxime ester in the photopolymerization reaction in combination with co-initiators (in multicomponent systems).

Future work will focus on the studies of the efficiency of oxime esters for the initiation of photopolymerization of meth(acrylate) compositions, both as one- and multicomponent systems. Additionally, the proposed procedures for the synthesis of oxime esters must comply with the principles and requirements of “Green Chemistry” as outlined by Anastas and Warner in 1990. Hence, further work should focus on developing synthesis methods aligned with this direction, i.e., without the need for a solvent or at room temperature.

## Figures and Tables

**Table 1:** Spectroscopic properties of oxime esters studied.

Dye	Solvent	λ_max_ [nm]	ε_max_ [M^−1^·cm^−1^]	ε_365 nm_ [M^−1^·cm^−1^]	λ_fl_ * [nm]	Stokes Shift [cm^−1^]	Ε_0→0_ [eV]
OE01	Et_2_O	328	24,597	1021	387	4648	3.48
	CHCl_3_	332	21,437	1973	393	4675	3.42
	AcOEt	328	23,604	1010	391	4912	3.47
	THF	330	22,175	1176	389	4596	3.50
	DCM	331	20,615	1581	395	4895	3.42
	ACE	324	15,039	656	392	5354	3.47
	MeOH	327	18,982	997	387	4741	3.48
	DMF	330	21,252	1519	402	5427	3.43
	MeCN	327	25,006	1386	398	5455	3.45
	DMSO	332	23,279	2260	406	5490	3.40
OE02	Et_2_O	328	2054	107	389	4781	3.48
	CHCl_3_	332	2242	216	395	4804	3.42
	AcOEt	328	2184	120	391	4912	3.47
	THF	330	2184	144	391	4728	3.50
	DCM	331	1959	169	396	4959	3.43
	ACE	329	2118	106	393	4950	3.47
	MeOH	328	1994	132	396	5235	3.45
	DMF	330	2129	179	401	5365	3.42
	MeCN	327	2107	122	398	5455	3.45
	DMSO	332	2172	231	404	5368	3.40
OE03	Et_2_O	330	3043	165	388	4530	3.47
	CHCl_3_	333	3047	386	395	4714	3.41
	AcOEt	330	3056	171	391	4728	3.45
	THF	331	3050	224	392	4701	3.44
	DCM	333	2577	276	396	4778	3.42
	ACE	324	3321	276	395	5548	3.45
	MeOH	329	2337	188	400	5395	3.42
	DMF	332	2757	292	402	5245	3.41
	MeCN	329	2840	306	399	5332	3.42
	DMSO	333	2837	416	408	5520	3.39
OE04	Et_2_O	330	3274	177	389	4596	3.47
	CHCl_3_	334	2863	359	395	4624	3.42
	AcOEt	330	3444	195	393	4858	3.45
	THF	332	2783	218	392	4610	3.46
	DCM	333	2328	254	397	4841	3.42
	ACE	327	2522	155	395	5265	3.46
	MeOH	330	3005	246	399	5240	3.42
	DMF	332	2739	302	402	5245	3.41
	MeCN	329	2550	290	399	5332	3.42
	DMSO	334	3017	440	406	5310	3.38
OE05	Et_2_O	328	3056	172	389	4781	3.47
	CHCl_3_	332	2901	289	394	4740	3.42
	AcOEt	328	2928	166	392	4978	3.46
	THF	330	3010	196	393	4858	3.50
	DCM	331	2700	268	394	4831	3.42
	ACE	323	4123	168	393	5514	3.47
	MeOH	328	2751	194	391	4912	3.43
	DMF	331	2925	263	401	5274	3.42
	MeCN	327	2606	279	398	5455	3.42
	DMSO	332	2562	334	405	5429	3.38
OE06	Et_2_O	305	4673	229	391	7211	3.39
	CHCl_3_	317	5213	235	395	6229	3.42
	AcOEt	309	5050	185	389	6656	3.46
	THF	310	4246	212	396	7006	3.50
	DCM	317	4799	223	395	6229	3.42
	ACE	323	7498	173	388	5187	3.47
	MeOH	314	4649	304	394	6466	3.43
	DMF	314	4290	251	398	5722	3.42
	MeCN	326	2749	620	389	4968	3.42
	DMSO	316	4640	419	399	6583	3.38

* λ_ex_ = 320 nm.

**Table 2:** Physical properties of the solvents.

No.	Solvent	Dimroth-Reichard Parameter	Kamlet—Taft Parameter
1	Et_2_O	34.5	0.27
2	CHCl_3_	39.1	0.58
3	AcOEt	38.1	0.55
4	THF	37.4	0.58
5	DCM	40.7	0.88
6	ACE	42.2	0.71
7	MeOH	55.5	0.6
8	DMF	43.2	1.03
9	MeCN	45.6	0.75
10	DMSO	45.1	1.00

**Table 3:** Summary of R^2^ parameters calculated for oxime esters.

Dye	Stokes Shift		λ_max ab_		ε_max_		ε_365 nm_		Ε_0→0_	
	D-R *	K-T *	D-R	K-T	D-R	K-T	D-R	K-T	D-R	K-T
OE01	0.0993	0.5588	0.0464	0.1065	0.1060	0.0298	0.0080	0.2809	0.0131	0.4989
OE02	0.4973	0.4678	0.0269	0.2151	0.1399	0.0002	0.0059	0.3295	0.1331	0.5174
OE03	0.5644	0.3677	0.0281	0.0809	0.4962	0.1031	0.0144	0.4071	0.2052	0.6263
OE04	0.5590	0.4240	0.0169	0.1217	0.0313	0.2795	0.0784	0.3491	0.2367	0.6172
OE05	0.1147	0.3472	0.0100	0.1144	0.0545	0.0465	0.0424	0.4689	0.1943	0.4706
OE06	0.1080	0.0712	0.1841	0.2563	0.0196	0.0100	0.1944	0.0899	0.0110	0.0440

* D-R—Dimroth-Reichardt scale, * K-T—Kamlet-Taft scale.

**Table 4:** Summary of R^2^ parameters of λ_fl_ calculated for oxime esters.

Dye	λ_fl_	
	D-R	K-T
OE01	0.0272	0.8320
OE02	0.3025	0.8351
OE03	0.4585	0.8518
OE04	0.4263	0.8893
OE05	0.0628	0.9009
OE06	0.0218	0.6270

**Table 5:** Fluorescence quantum yields of oxime esters in solvent of different polarity.

Dye	Φ_fl_									
	Et_2_O	CHCl_3_	AcOEt	THF	DCM	ACE	MeOH	DMF	MeCN	DMSO
OE01	0.1140	0.0944	0.1069	0.1488	0.0841	0.1044	0.0963	0.0934	0.1036	0.0541
OE02	0.1343	0.1269	0.1211	0.1713	0.1029	0.1317	0.1064	0.1174	0.1201	0.0660
OE03	0.1327	0.1035	0.1192	0.1667	0.0929	0.1276	0.1021	0.1030	0.1132	0.0507
OE04	0.1189	0.1100	0.1280	0.1681	0.1017	0.1212	0.1086	0.1125	0.1188	0.0520
OE05	0.1284	0.1295	0.1365	0.1561	0.1175	0.1246	0.1404	0.1268	0.1239	0.0741
OE06	0.1387	0.1637	0.1442	0.1666	0.1316	0.1329	0.1136	0.1545	0.1222	0.0592

**Table 7:** Rate of photodecay of the oxime esters obtained.

Photoinitiator	Rate Constants of Photodecay |−k_1_|
OE01	6.09 × 10^−3^
OE02	4.53 × 10^−3^
OE03	6.07 × 10^−3^
OE04	9.23 × 10^−3^
OE05	6.45 × 10^−3^
OE06	0.27 × 10^−3^

**Table 8:** Effect of photopolymerization process conditions on the degree of monomer conversion and induction time for aliphatic substituted oxime ester (OE02).

Light Intensity [mW/cm^2^]	Concentration of Photoinitiator									
	0.1%		0.2%		0.5%		1%		2%	
	C_FTIR_ [%]	t_ind_ [s]	C_FTIR_ [%]	t_ind_ [s]	C_FTIR_ [%]	t_ind_ [s]	C_FTIR_ [%]	t_ind_ [s]	C_FTIR_ [%]	t_ind_ [s]
1.0	45.50	6.89	45.00	7.42	68.00	2.64	71.00	1.04	72.00	1.04
12.7	46.35	3.18	50.00	3.17	69.00	1.05	71.00	1.04	73.00	0.00
19.1	50.50	2.13	50.00	1.06	71.00	1.05	71.00	0.00	74.00	0.00
27.7	49.00	1.59	55.50	1.06	71.00	1.05	72.00	0.00	74.50	0.00

**Table 9:** Effect of photopolymerization process conditions on the degree of monomer conversion and induction time for aromatic substituted oxime ester (OE03).

Light Intensity [mW/cm^2^]	Concentration of Photoinitiator									
	0.1%		0.2%		0.5%		1%		2%	
	C_FTIR_ [%]	t_ind_ [s]	C_FTIR_ [%]	t_ind_ [s]	C_FTIR_ [%]	t_ind_ [s]	C_FTIR_ [%]	t_ind_ [s]	C_FTIR_ [%]	t_ind_ [s]
1.0	13.00	11.67	36.00	7.95	38.00	5.30	43.00	4.24	49.50	2.66
12.7	29.00	3.18	29.50	3.18	48.50	1.59	49.00	1.59	58.00	1.06
19.1	37.50	1.59	32.00	1.59	46.00	1.59	54.00	1.06	57.50	0.53
27.7	34.50	1.59	40.00	0.53	51.00	0.53	50.00	0.53	61.00	0.53

**Table 10:** Photopolymerization reaction rate of selected examples.

Light Intensity [mW/cm^2^]	Photoinitiator Concentration			
	0.1%		2%	
	OE02	OE03	OE02	OE03
1.0	0.0386	0.0100	0.1855	0.0938
12.7	0.1155	0.0713	0.2259	0.1500
19.1	0.1368	0.1110	0.2388	0.1310
27.7	0.1571	0.1019	0.2614	0.1503

## Data Availability

The original contributions presented in this study are included in the article/Supplementary Materials. Further inquiries can be directed to the corresponding authors.

## Supplementary Materials

The following supporting information can be downloaded at: https://www.mdpi.com/article/10.3390/ma19030558/s1, Figure S1: ^1^H NMR spectrum of A2B, Figure S2: ^1^H NMR spectrum of O2B, Figure S3: ^1^H NMR spectrum of OE01 (solvent—DMSO-d6), Figure S4: ^13^C NMR spectrum of OE01 (solvent—DMSO-d6), Figure S5: ^1^H NMR spectrum of OE02 (solvent—DMSO-d6), Figure S6: ^13^C NMR spectrum of OE02 (solvent—DMSO-d6), Figure S7: ^1^H NMR spectrum of OE03 (solvent—DMSO-d6), Figure S8: ^13^C NMR spectrum of OE03 (solvent—DMSO-d6), Figure S9: ^1^H NMR spectrum of OE04 (solvent—DMSO-d6), Figure S10: ^13^C NMR spectrum of OE04 (solvent—DMSO-d6), Figure S11: ^1^H NMR spectrum of OE05 (solvent—DMSO-d6), Figure S12: ^13^C NMR spectrum of OE05 (solvent—DMSO-d6), Figure S13: ^1^H NMR spectrum of OE06 (solvent—DMSO-d6), Figure S14: ^13^C NMR spectrum of OE06 (solvent—DMSO-d6), Figure S15: FT-IR spectrum of OE01, Figure S16: FT-IR spectrum of OE02, Figure S17: FT-IR spectrum of OE03, Figure S18: FT-IR spectrum of OE04, Figure S19: FT-IR spectrum of OE05, Figure S20: FT-IR spectrum of OE06, Figure S21: Normalized absorption spectra of oxime esters in acetone (ACE) recorded at room temperature, Figure S22: Normalized absorption spectra of oxime esters in ethyl acetate (AcOEt) recorded at room temperature, Figure S23: Normalized absorption spectra of oxime esters in chloroform (CHCl_3_) recorded at room temperature, Figure S24: Normalized absorption spectra of oxime esters in dichloromethane (DCM) recorded at room temperature, Figure S25: Normalized absorption spectra of six oxime esters in *N*,*N*-dimethylformamide (DMF) recorded at room temperature, Figure S26: Normalized absorption spectra of oxime esters in dimethyl sulfoxide (DMSO) recorded at room temperature, Figure S27: Normalized absorption spectra of oxime esters in diethyl ether (Et_2_O) recorded at room temperature, Figure S28: Normalized absorption spectra of oxime esters in acetonitrile (MeCN) recorded at room temperature, Figure S29: Normalized absorption spectra of oxime esters in methanol (MeOH) recorded at room temperature, Figure S30: Normalized absorption spectra of oxime esters in tetrahydrofuran (THF) recorded at room temperature, Figure S31: Normalized fluorescence spectra of OE01 in different solvents recorded at room temperature, Figure S32: Normalized fluorescence spectra of OE02 in different solvents recorded at room temperature, Figure S33: Normalized fluorescence spectra of OE03 in different solvents recorded at room temperature, Figure S34: Normalized fluorescence spectra of OE04 in different solvents recorded at room temperature, Figure S35: Normalized fluorescence spectra of OE05 in different solvents recorded at room temperature, Figure S36: Normalized fluorescence spectra of OE06 in different solvents recorded at room temperature, Figure S37: Photolysis of OE01 in MeCN under @LED365 nm with a light intensity (50 mW/cm^2^), Figure S38: Photolysis of OE02 in MeCN under @LED365 nm with a light intensity (50 mW/cm^2^), Figure S39: Photolysis of OE03 in MeCN under @LED365 nm with a light intensity (50 mW/cm^2^), Figure S40: Photolysis of OE04 in MeCN under @LED365 nm with a light intensity (50 mW/cm^2^), Figure S41: Photolysis of OE05 in MeCN under @LED365 nm with a light intensity (50 mW/cm^2^), Figure S42: Photolysis of OE06 in MeCN under @LED365 nm with a light intensity (50 mW/cm^2^).
