# Supplementary material for: Synthesis, Properties and Application of Novel 2-Substituted Benzothiazole-Based Oxime Esters
Source: Materials (Basel). 2026 Jan 30;19(3):558. doi: 10.3390/ma19030558 (PMC12898230; doi:10.3390/ma19030558)
Supplement: Supplementary file 1 [file materials-19-00558-s001.zip › materials-3976044-supplementary.pdf]

materials

Supplementary Materials

Synthesis, properties and application of novel 2-substituted benzothiazole-based oxime esters

Monika Dzwonkowska-Zarzycka*, Alicja Balcerak-Woźniak*, Janina Kabatc-Borcz

Department of Organic Chemistry, Faculty of Chemical Technology and Engineering, Bydgoszcz University of Science and Technology, Seminaryjna 3, 85-326 Bydgoszcz, Poland
* Correspondence: monika.dzwonkowska-zarzycka@pbs.edu.pl (M.D.-Z.); alicja.balcerak@pbs.edu.pl (A.B.-W.)

1. Synthesis protocols

1.1. Synthesis of A2B – 4-(2-benzothiazol)benzaldehyde

A mixture of 2-aminobenzenethiol (0.428 ml, 4 mmol), terephalaldehyde (0.804 g, 6 mmol) and $Na_2S_2O_5$ (0.761g, 4 mmol) was dissolved in DMF (25 ml) and refluxed overnight at 110℃. When the reaction was completed, the mixture was poured into water and pale yellow solid was precipitated. The obtained product (light yellow color) was extracted with a DCM/H_2O (1/1 v/v) mixture for several times. The organic layer was dried with the addition of $MgSO_4$. Then the solvent was evaporated and orange solid was obtained [1].

1.2. Synthesis of O2B - 4-(1,3-benzothiazol-2-yl)benzaldehyde oxime

4-(2-Benzothiazol)benzaldehyde (2.54 g, 10 mmol) and hydroxylamine hydrochloride (1.04 g, 15 mmol) were placed into round bottom flask. Then 100 ml of 50% aqueous solution of methanol and 6 ml of sodium cabonate aqueous solution (0.75 M) were added. The mixture was stirred at room temperature for 5 hours. The product was used for further synthesis steps (without further purification) [2].

1.3. Synthesis of oxime esters

4-(1,3-Benzothiazol-2-yl)benzaldehyde oxime (0.01 mol) was dissolved in dry acetone (20 ml) and then triethylamine (TEA) (0.015 mol) was added dropwise to the solution at 0°C. The reaction mixture was stirred for 5 min at 0°C and next for 6h at room temperature. Then, appropriate acid chloride (0.01 mol) was added and the mixture was stirred at 0°C for 10-30 min. After evaporation of the residue, the product was washed with cold ether (5 ml), hot water and then purified by column chromatography on silica gel eluting with chloroform or/and crystallized from the appropriate solvent.

Academic Editor(s): Name

Received: date
Revised: date
Accepted: date
Published: 30 January 2026

Copyright: © 2026 by the authors. Licensee MDPI, Basel, Switzerland. This article is an open access article distributed under the terms and conditions of the Creative Commons Attribution (CC BY) license.

2. NMR spectra

A2B 4-(2-benzothiazol)benzaldehyde

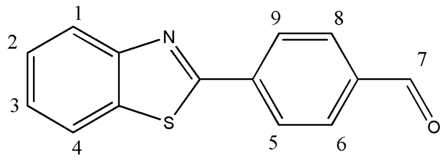

^{1}H NMR (400 MHz, $CDCl_3$-d6), δ (ppm): 10.03 (s, 1H, H-7); 8.23 – 8.21 (d, 2H, H-8, H-6), 8.08 – 8.06 (d, 1H, H-1), 7.96 – 7.94 (d, 2H, H-9, H-5), 7.90 – 7.88 (d, 1H, H-4) 7.05 – 7.46 (t, 1H, H-3), 7.41 – 7,37 (t, 1H, H-2)

Molecular weight – 239.29 g/mol

Melting point – 131.3 °C

Pale yellow powder – 64%

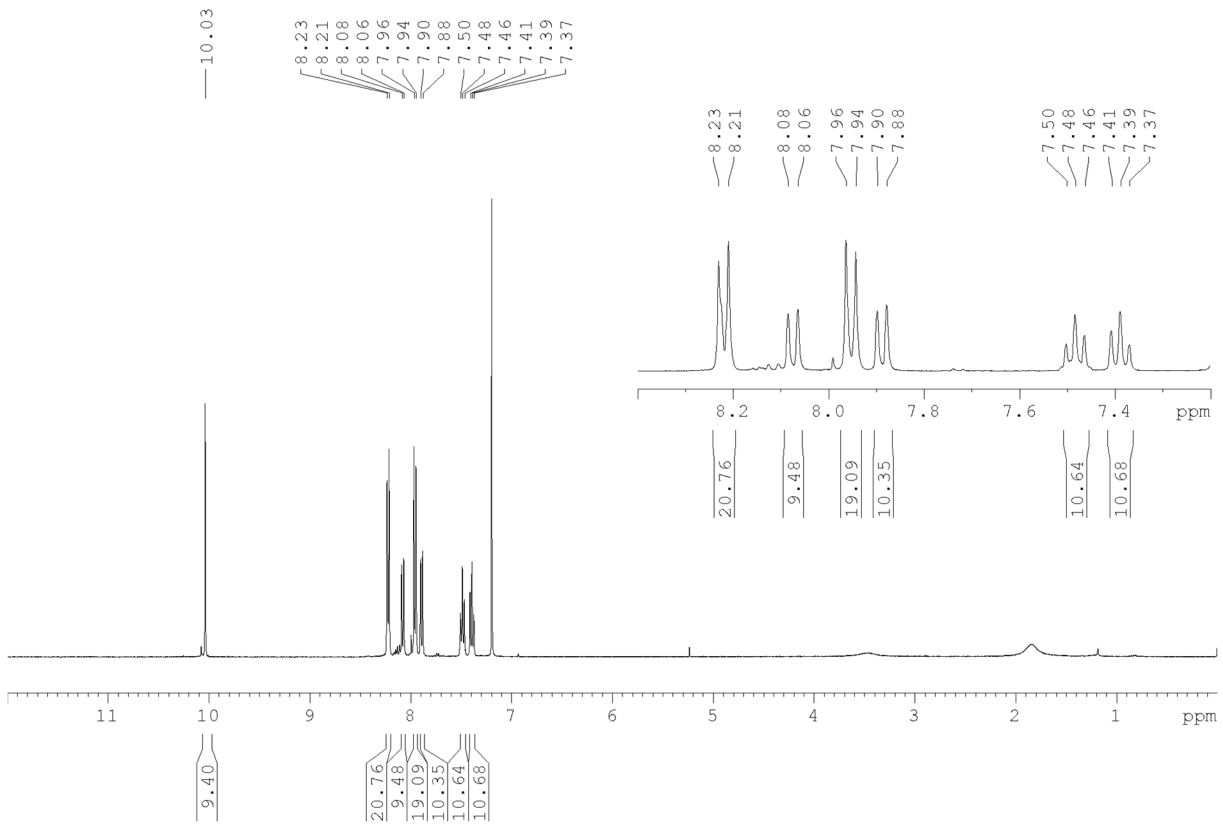

Figure S1. ^{1}H NMR spectrum of A2B.

O2B 4-(1,3-benzothiazol-2-yl)benzaldehyde oxime

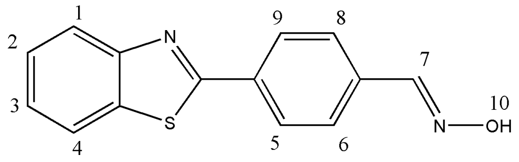

^{1}H NMR (400 MHz, DMSO–d6), δ (ppm): 11,5471 (s, 1H, H-10); 8,2505 (s, 1H, H-7), 8,1838 – 8,1640 (d, 1H, H-1), 8,1476-8,1268 (d, 2H, H-8, H-6), 8,0929 – 8,0727 (d, 1H, H-4) 7,8054 – 7,7845 (d, 2H, H-5, H-9), 7,5897 – 7,5486 (t, 1H, H-3), 7,5062 – 7,4658 (t, 1H, H-2)

Molecular weight – 254.31 g/mol

Melting point – 213.2 °C

Pale yellow powder

Yield – 76 %

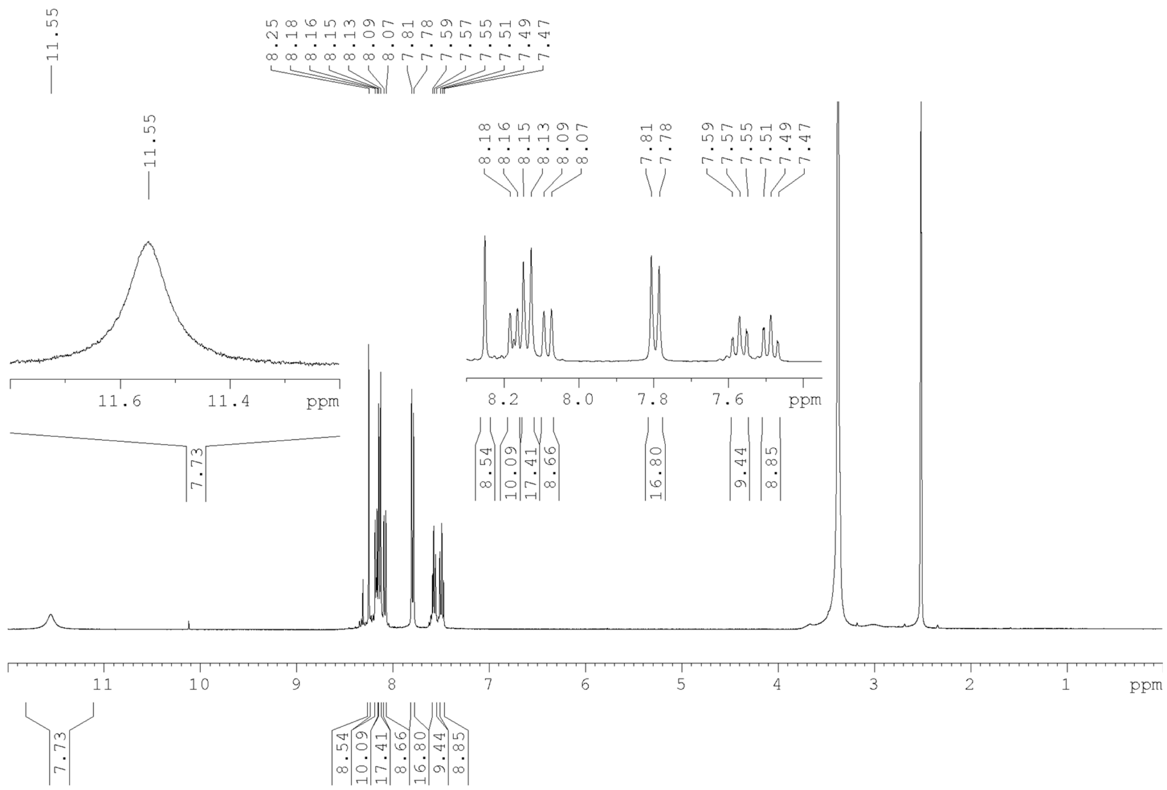

Figure S2. ^{1}H NMR spectrum of O2B.

[4-(1,3-benzothiazol-2-yl)phenyl]methyleneamino]acetate (OE01)

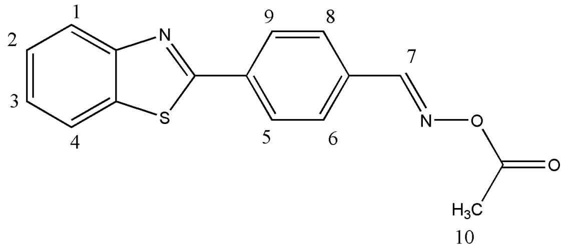

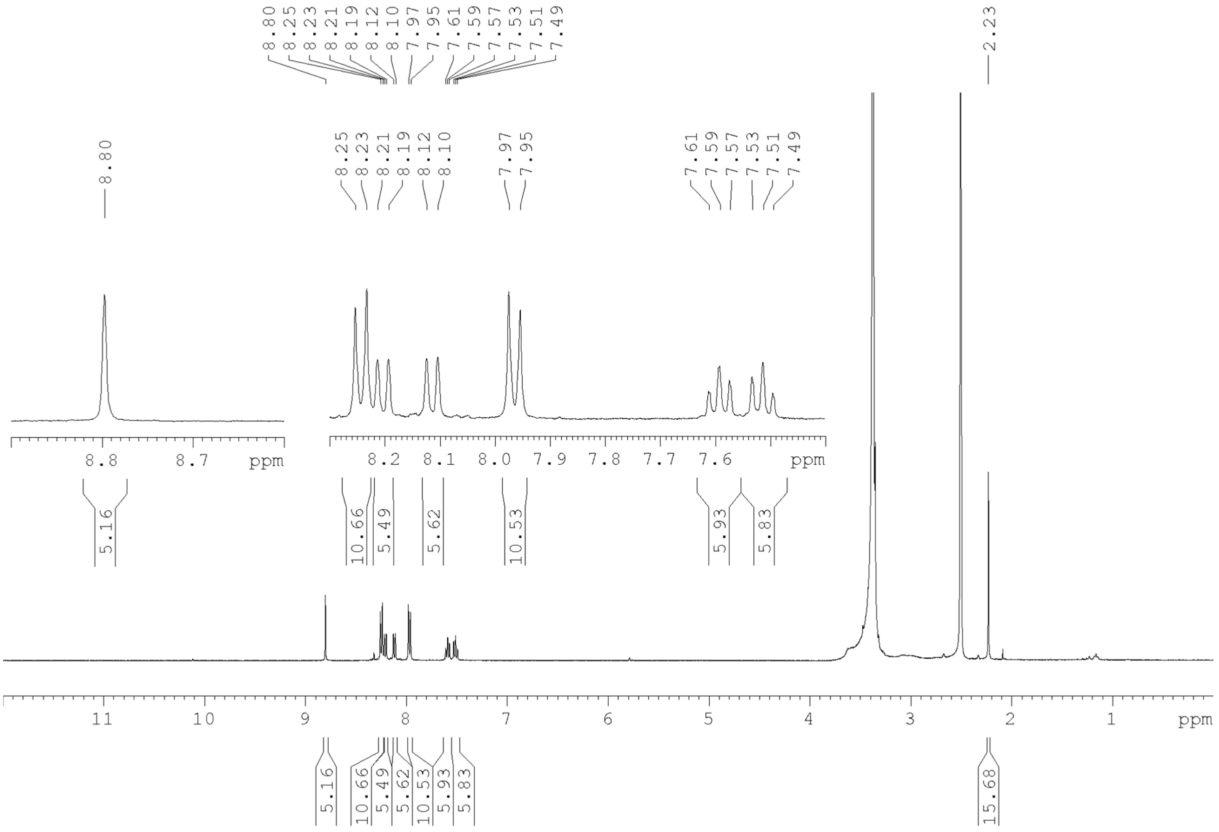

Figure S3. ^{1}H NMR spectrum of OE01 (solvent - DMSO-d6).

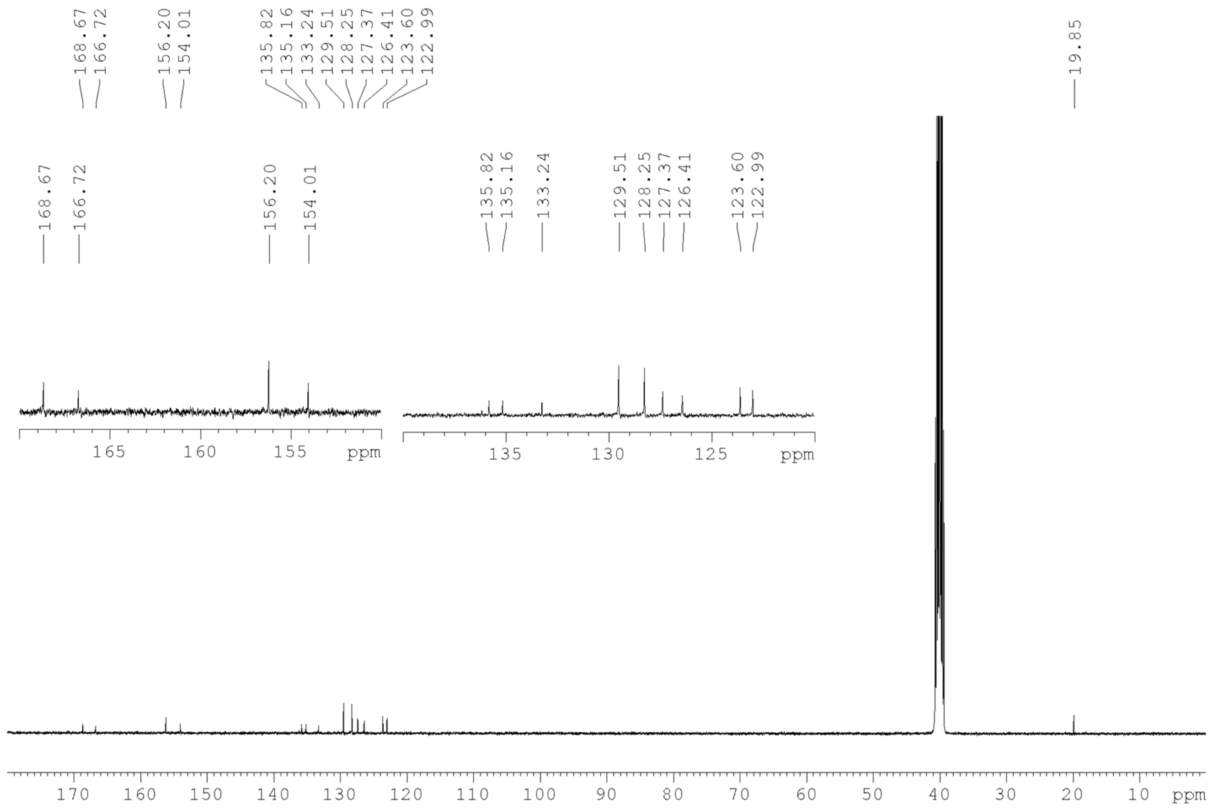

Figure S4. ^{13}C NMR spectrum of OE01 (solvent - DMSO-d6).

[4-(1,3-benzothiazol-2-yl)phenyl]methyleneamino]propanate (OE02)

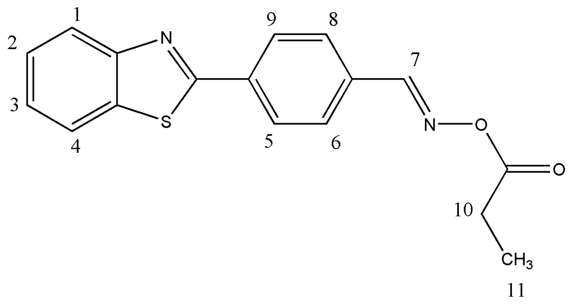

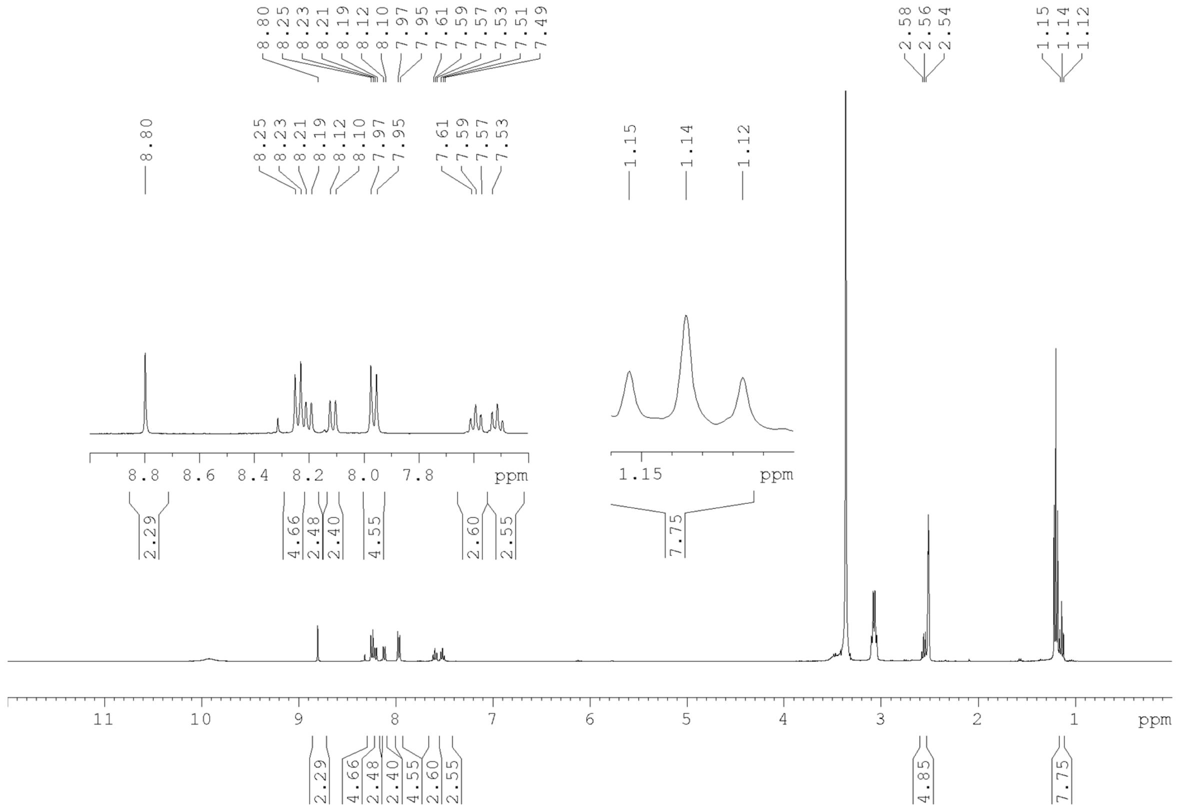

Figure S5. ^{1}H NMR spectrum of OE02 (solvent - DMSO-d6).

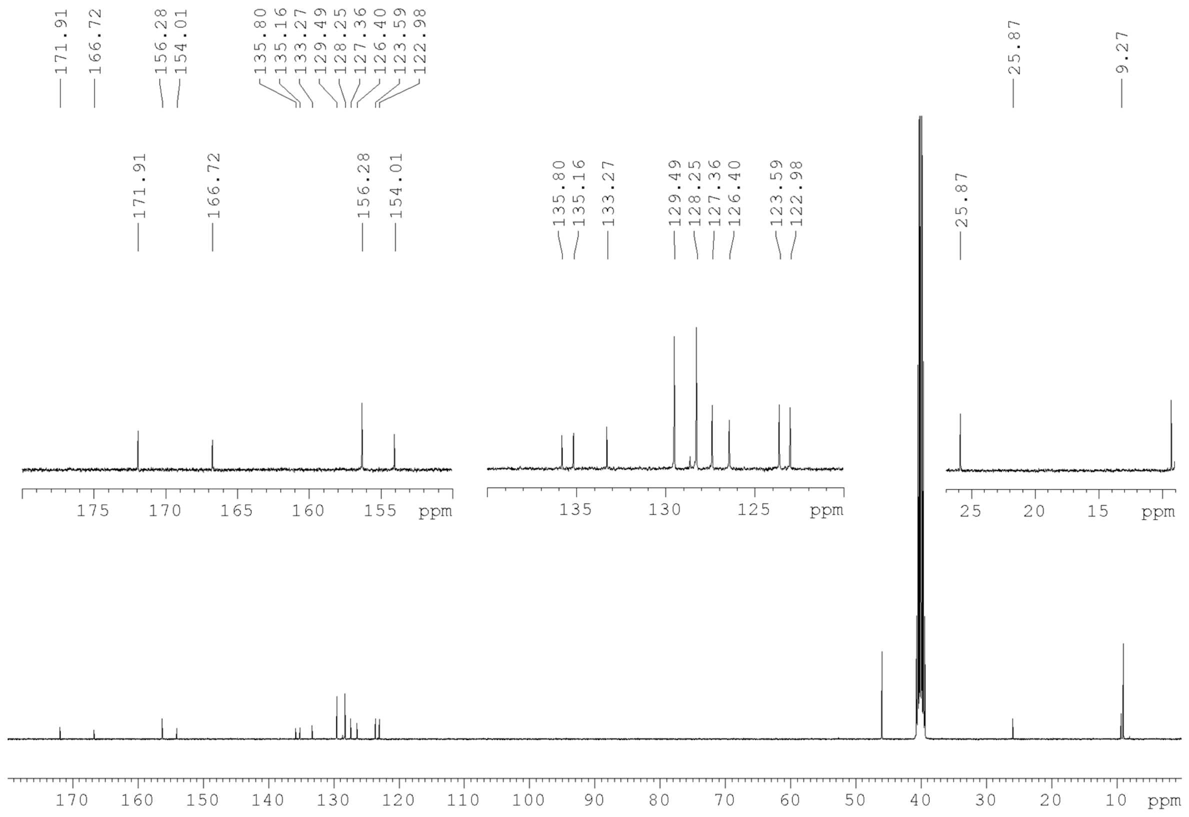

Figure S6. ^{13}C NMR spectrum of OE02 (solvent - DMSO-d6).

[4-(1,3-benzothiazol-2-yl)phenyl]methyleneamino]benzoate (OE03)

https://doi.org/10.3390/ma19030558

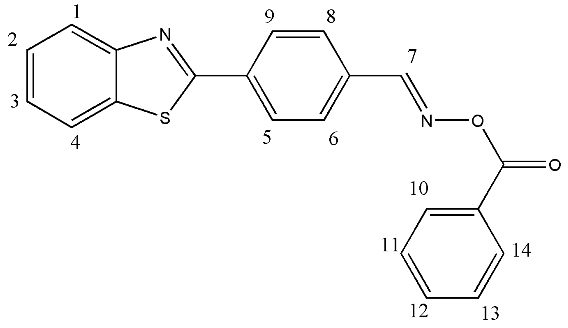

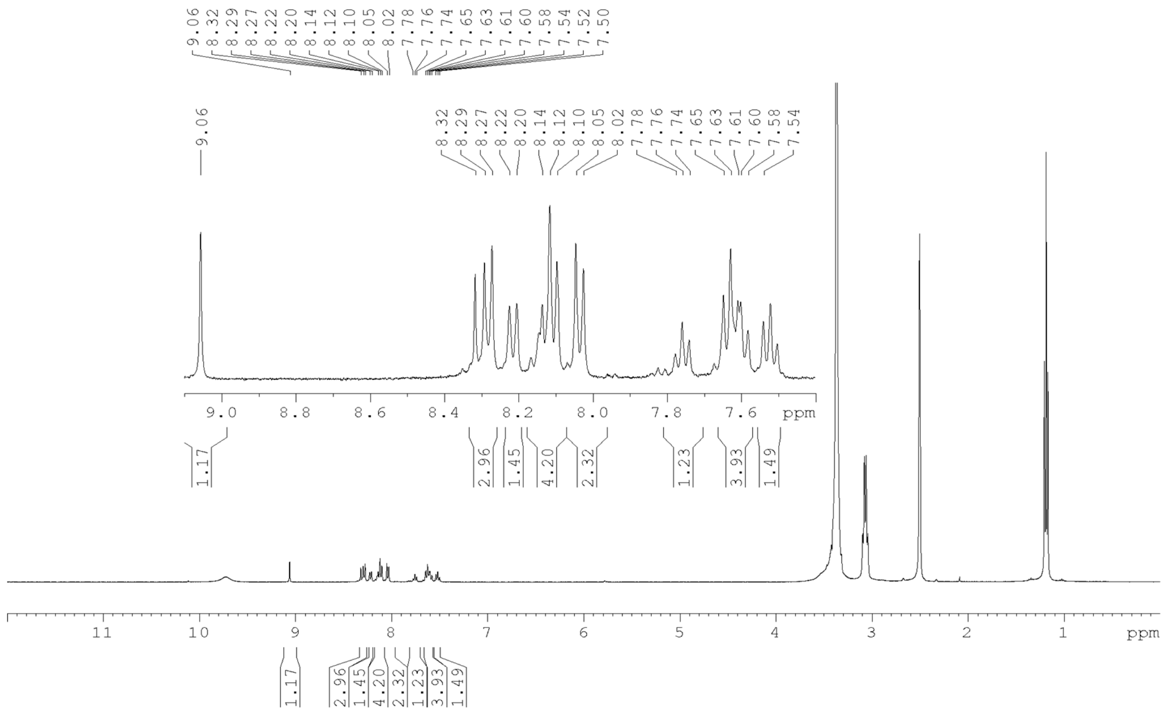

Figure S7. ^{1}H NMR spectrum of OE03 (solvent - DMSO-d6).

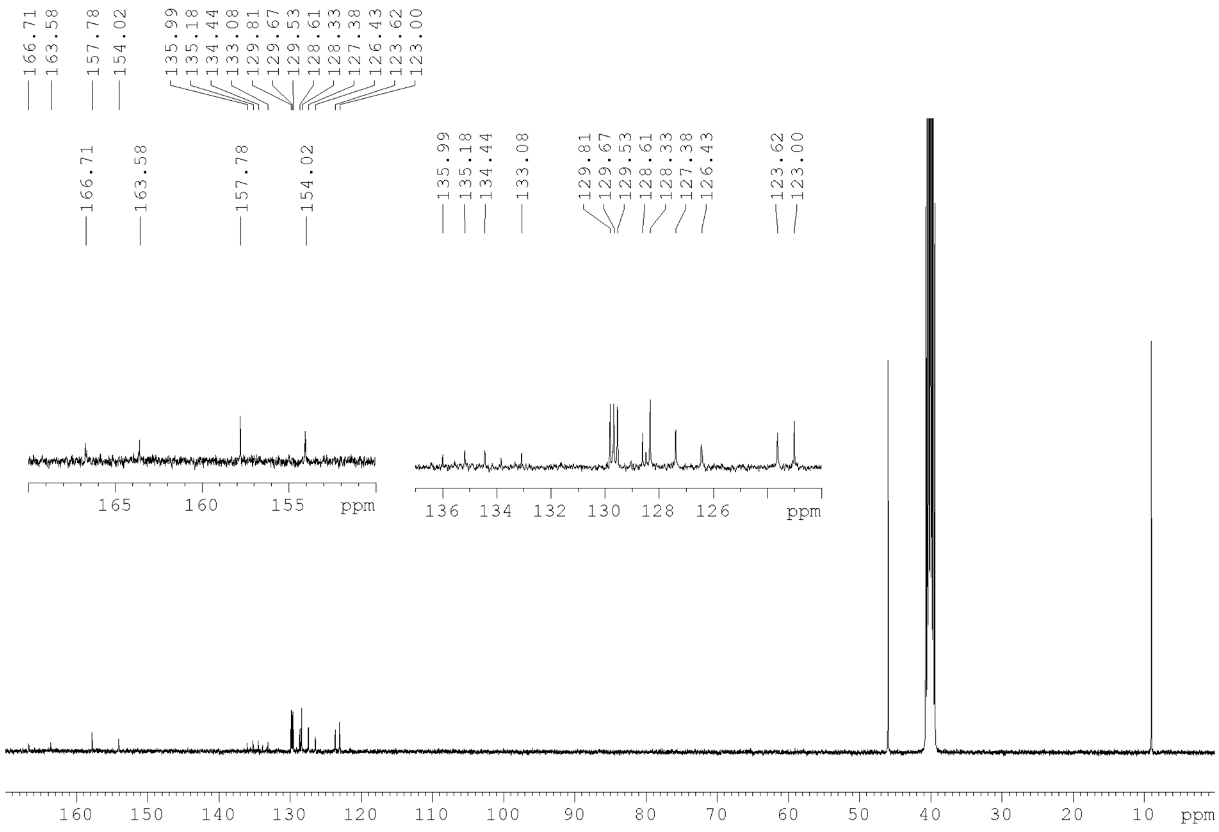

Figure S8. ^{13}C NMR spectrum of OE03 (solvent - DMSO-d6).

[4-(1,3-benzothiazol-2-yl)phenyl]methyleneamino]-4-methylbenzoate (OE04)

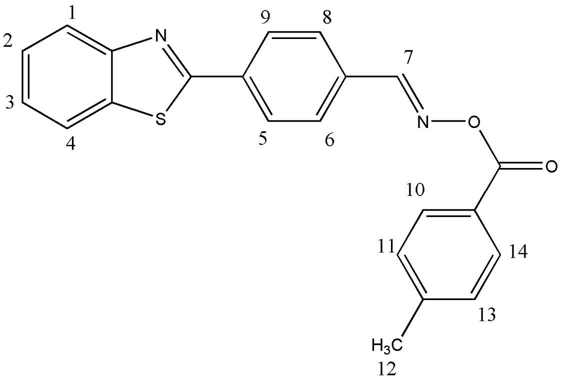

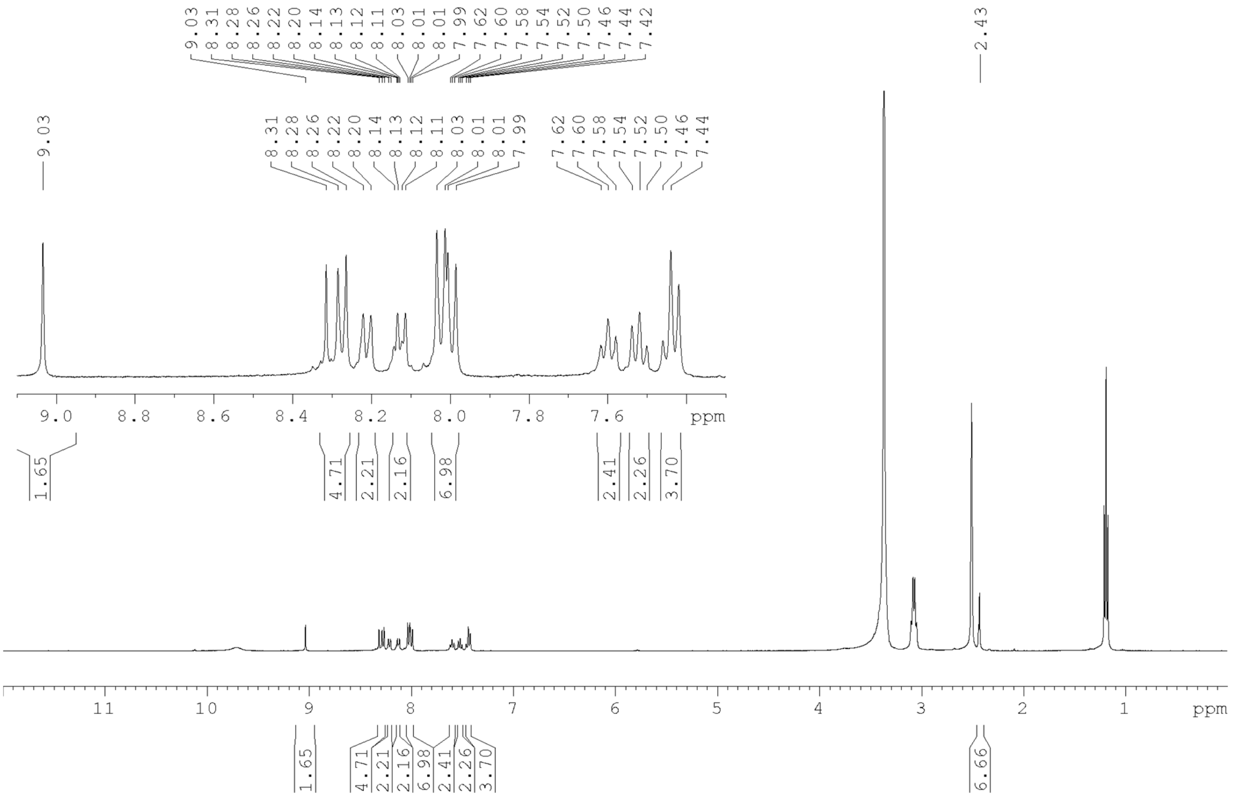

Figure S9. ^{1}H NMR spectrum of OE04 (solvent - DMSO-d6).

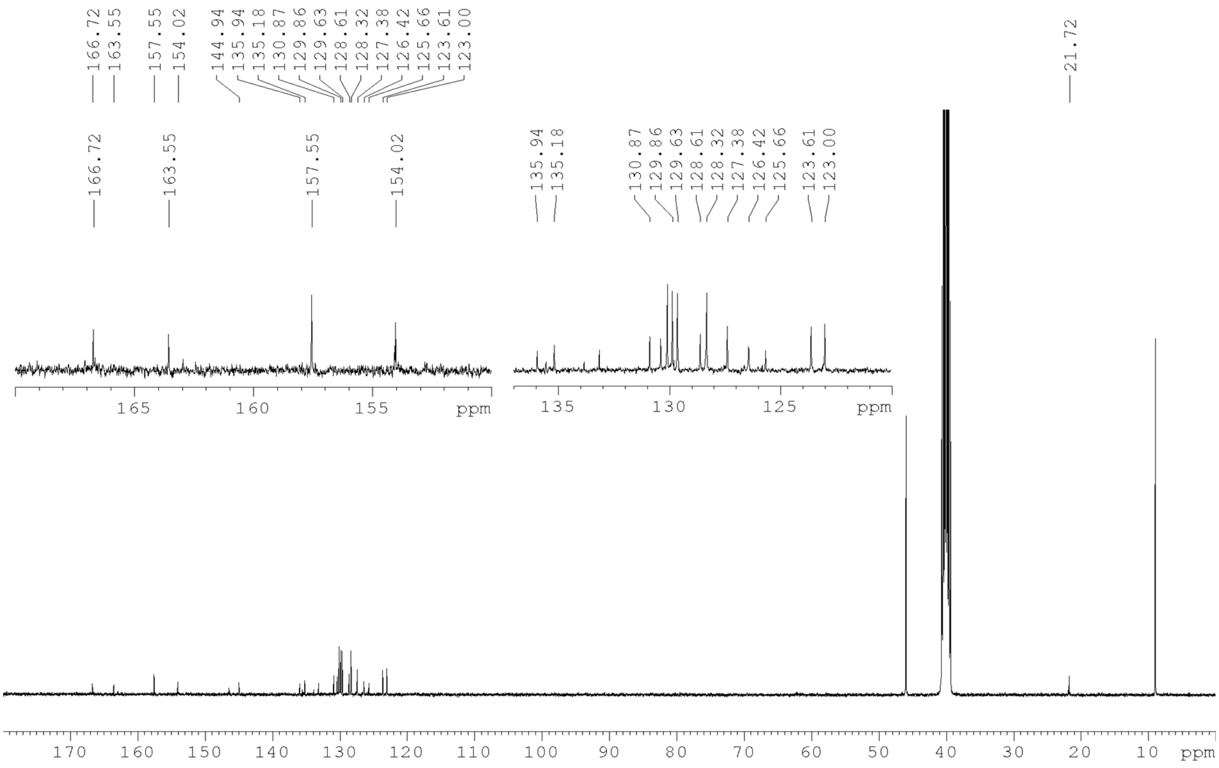

Figure S10. ^{13}C NMR spectrum of OE04 (solvent - DMSO-d6).

[4-(1,3-benzothiazol-2-yl)phenyl]methyleneamino]dodecanoate (OE05)

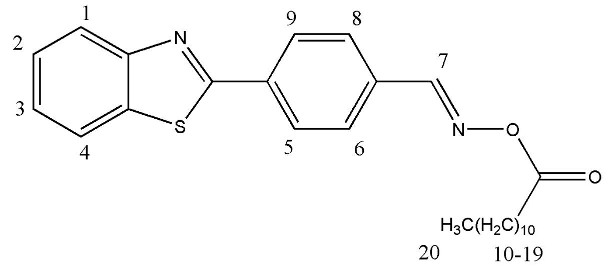

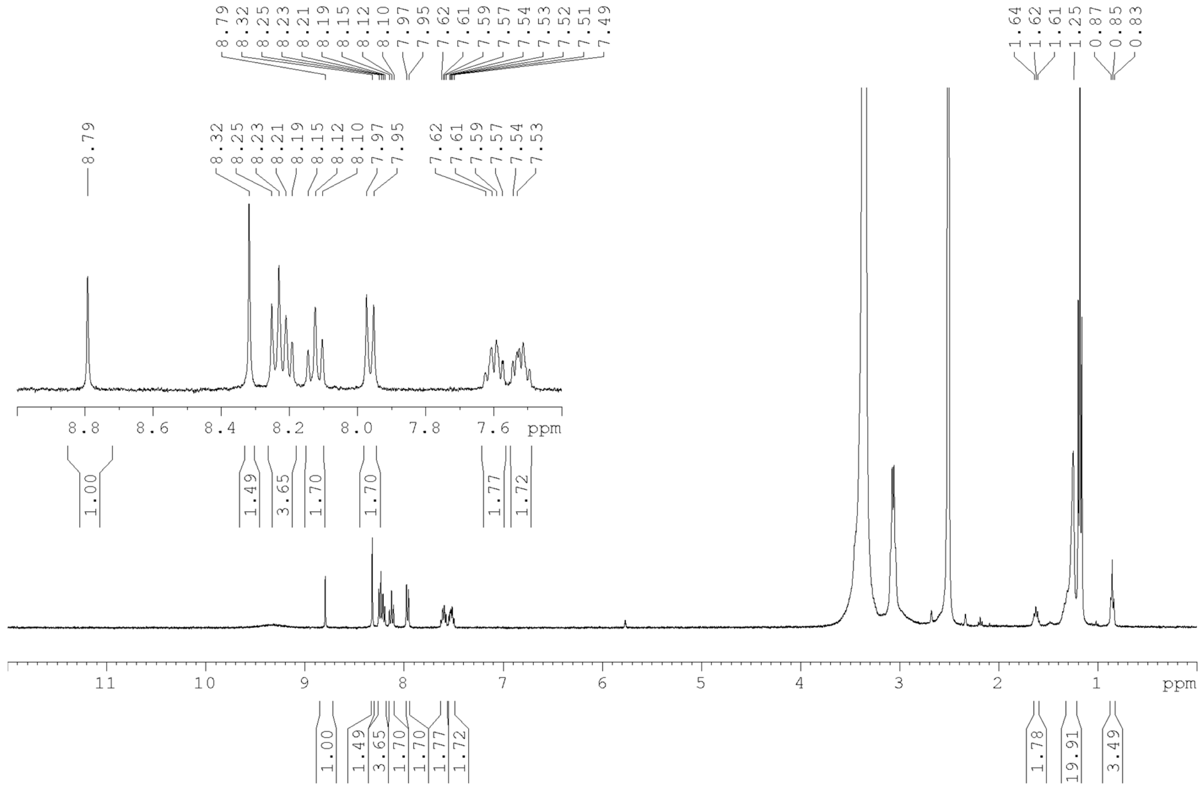

Figure S11. ^{1}H NMR spectrum of OE05 (solvent - DMSO-d6).

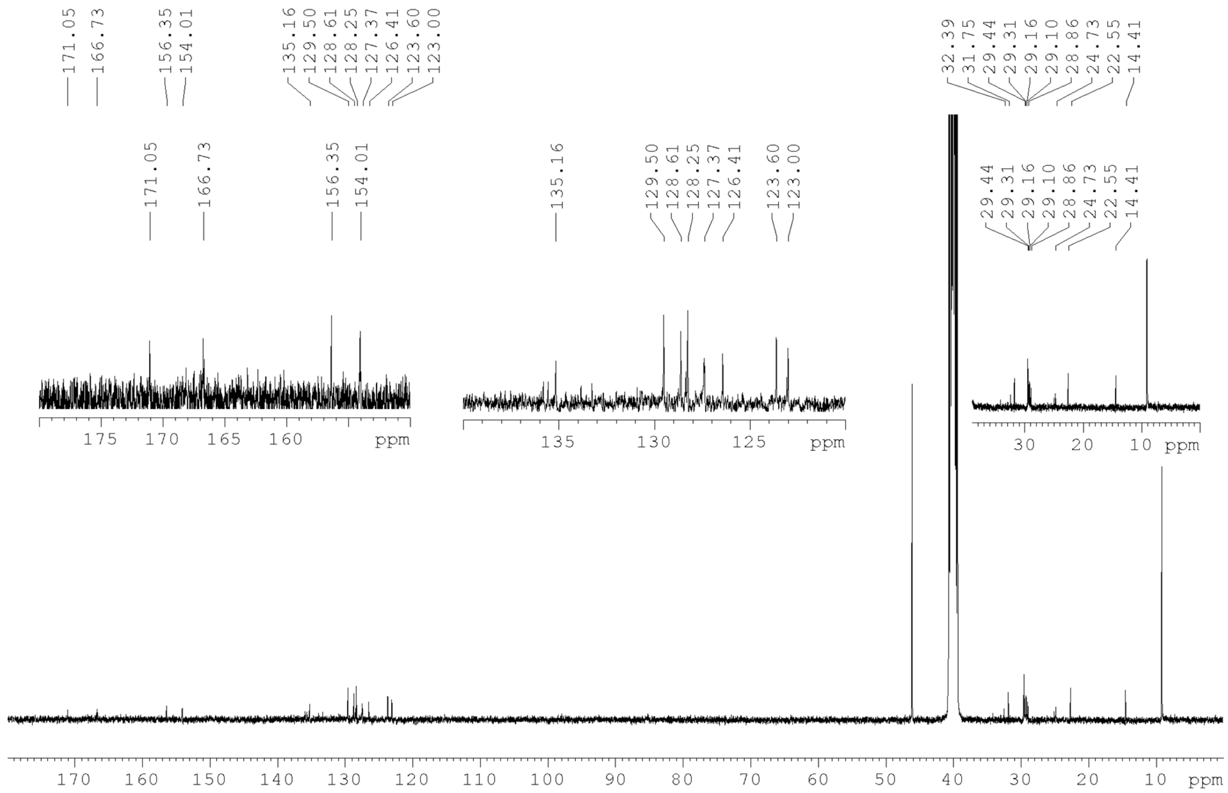

Figure S12. ^{13}C NMR spectrum of OE05 (solvent - DMSO-d6).

[4-(1,3-benzothiazol-2-yl)phenyl]methyleneamino]-4-(dimethylamino)benzoate (OE06)

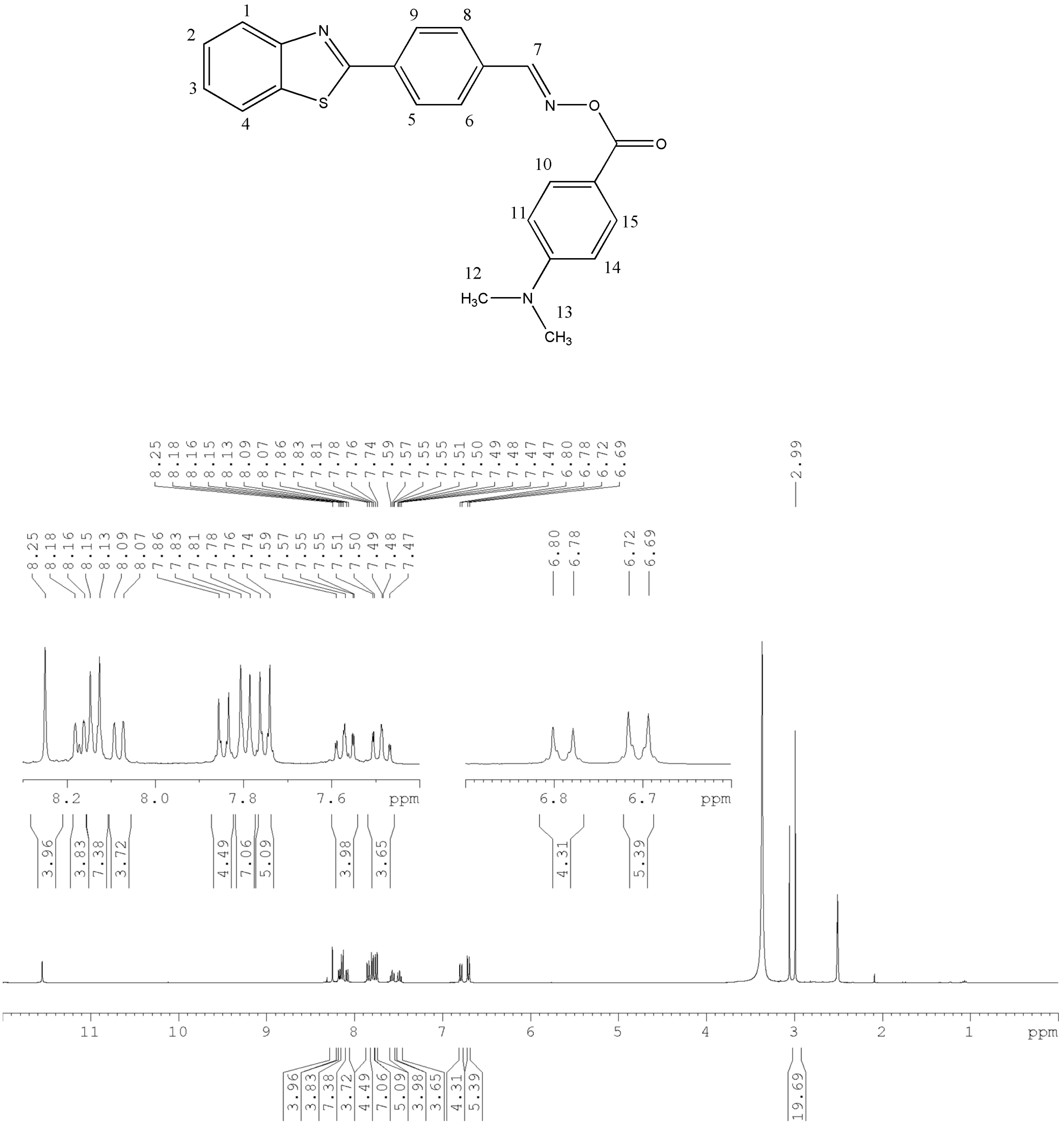

Figure S13. ^{1}H NMR spectrum of OE06 (solvent - DMSO-d6).

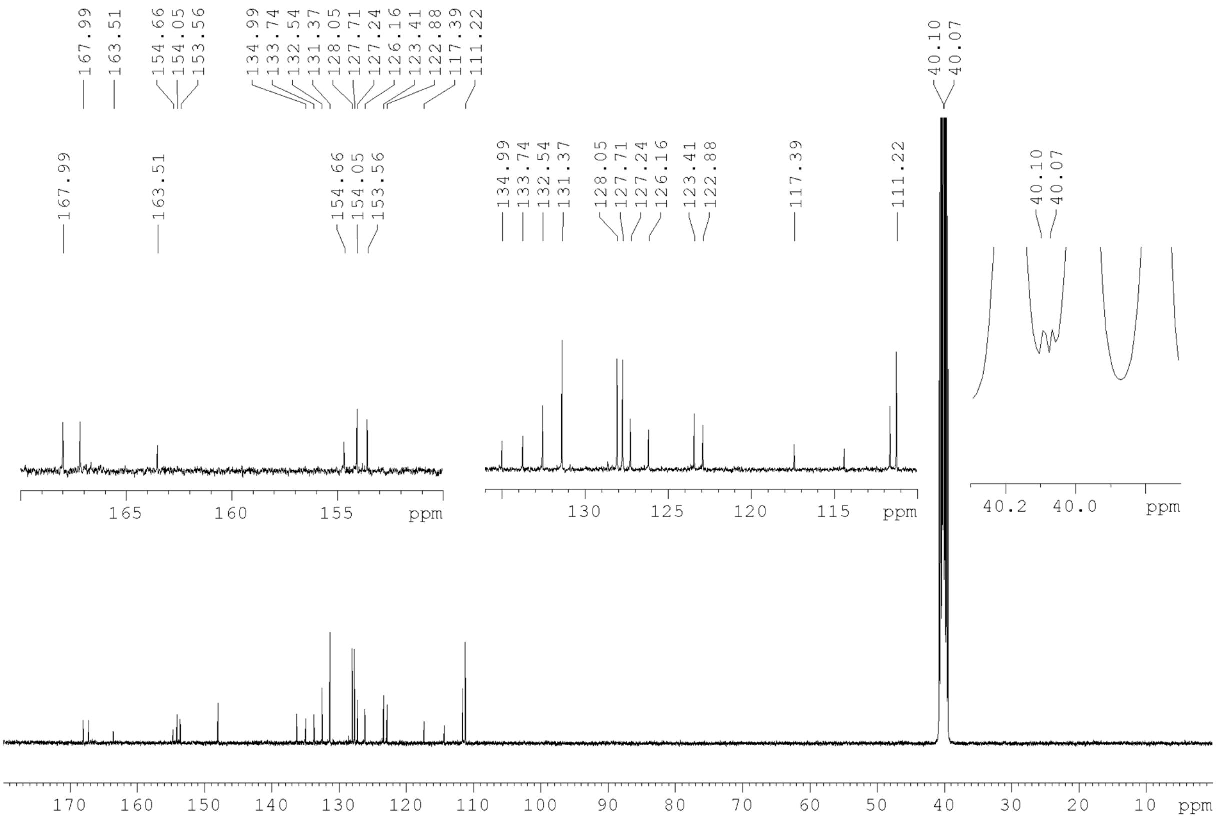

Figure S14. ^{13}C NMR spectrum of OE06 (solvent - DMSO-d6).

3. FT-IR spectra

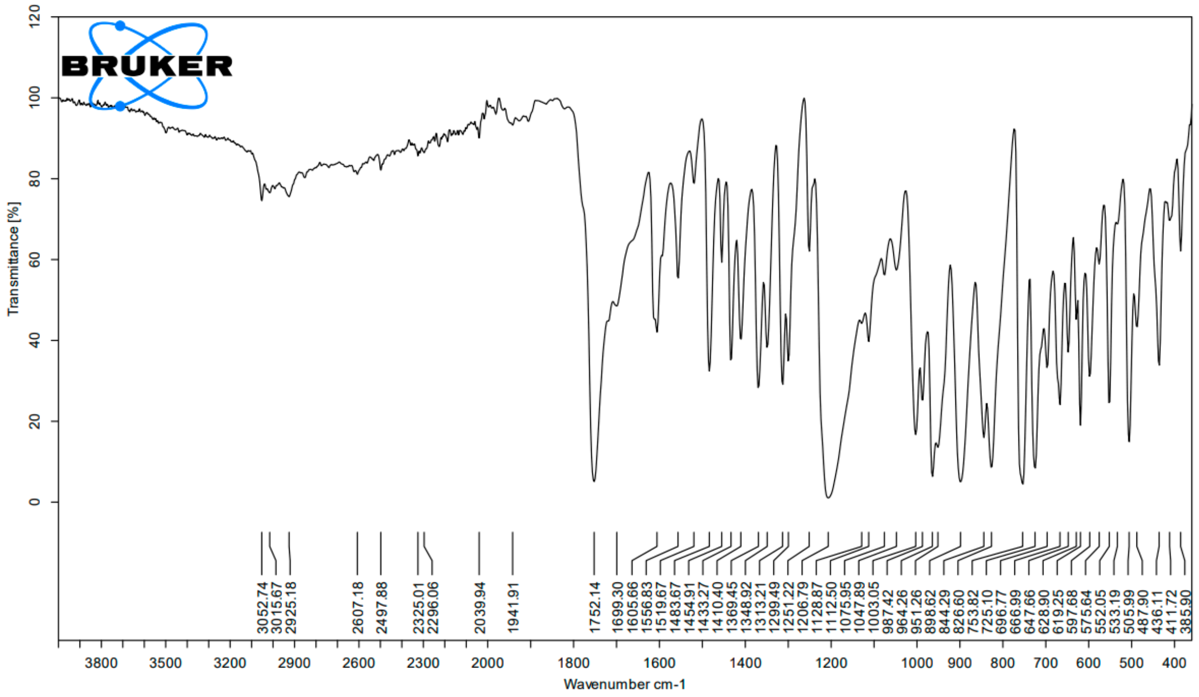

Figure S15. FT-IR spectrum of OE01.

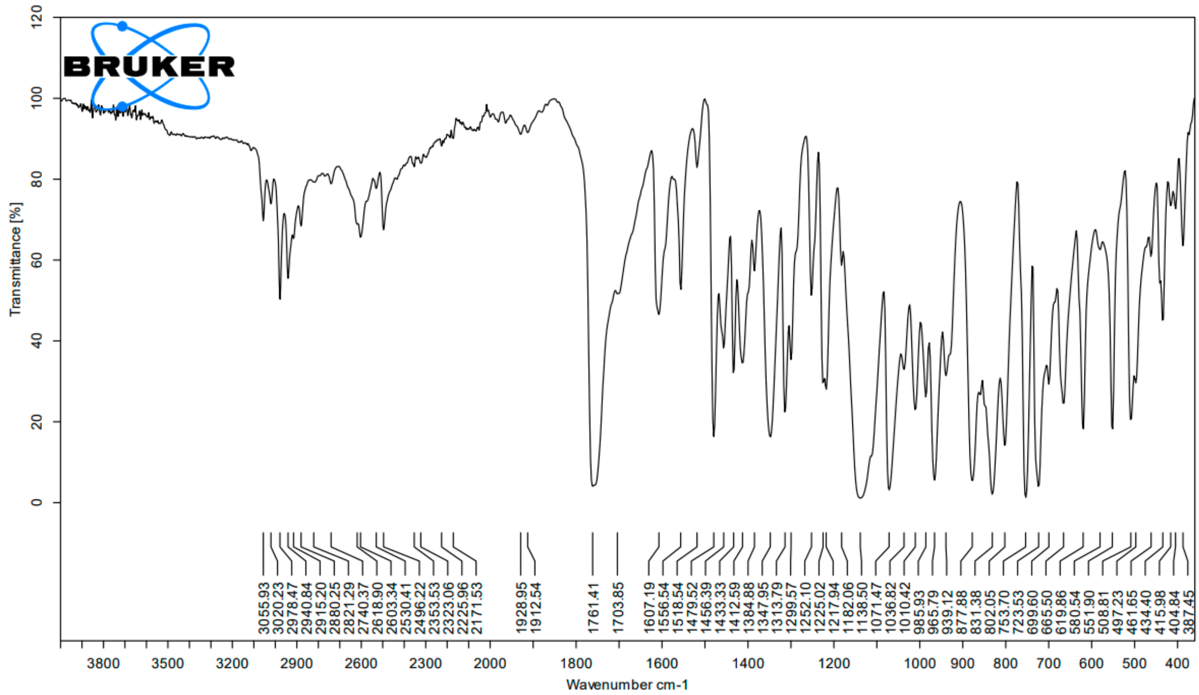

Figure S16. FT-IR spectrum of OE02.

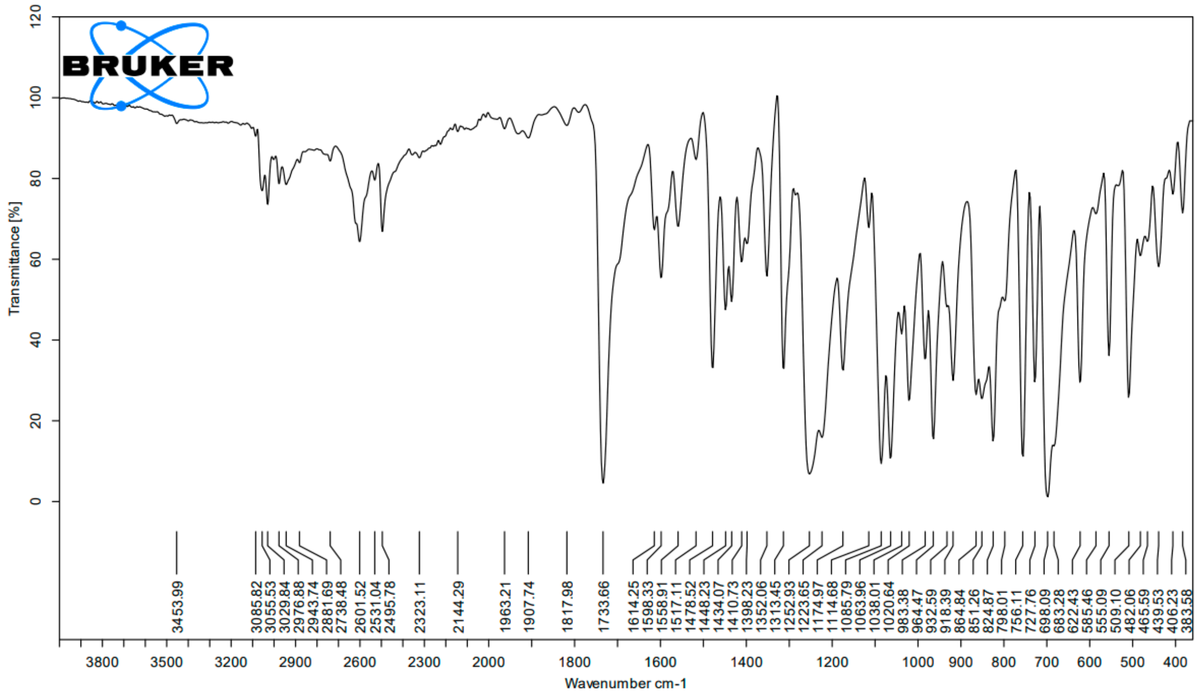

Figure S17. FT-IR spectrum of OE03.

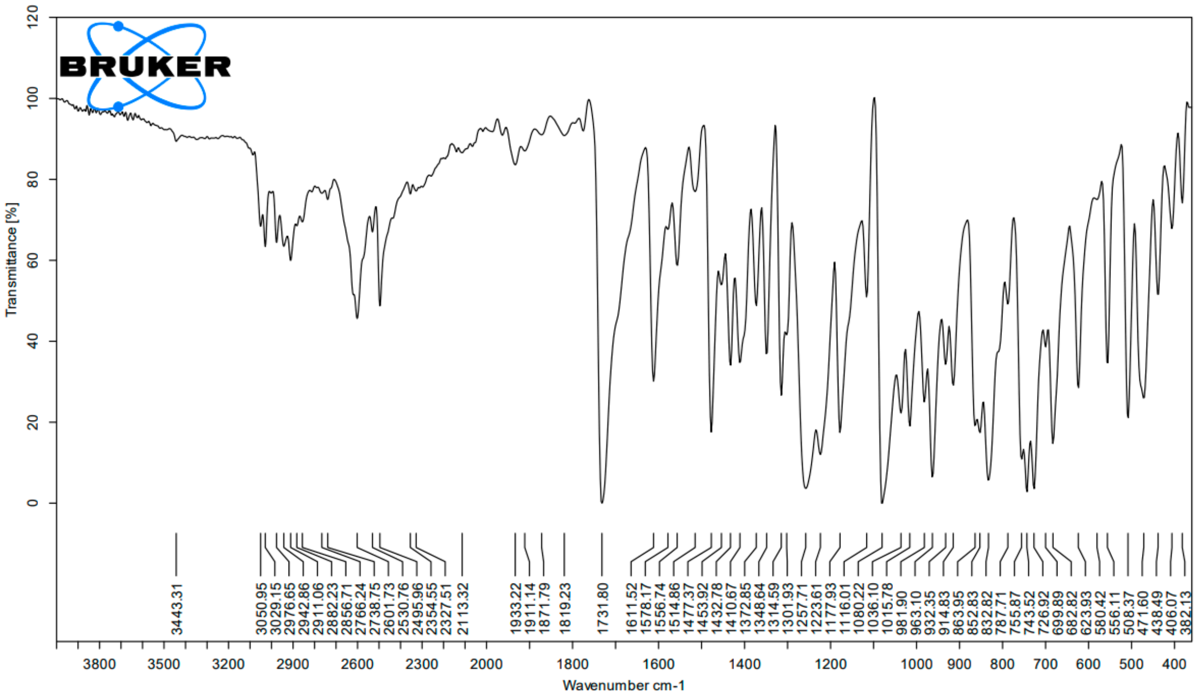

Figure S18. FT-IR spectrum of OE04.

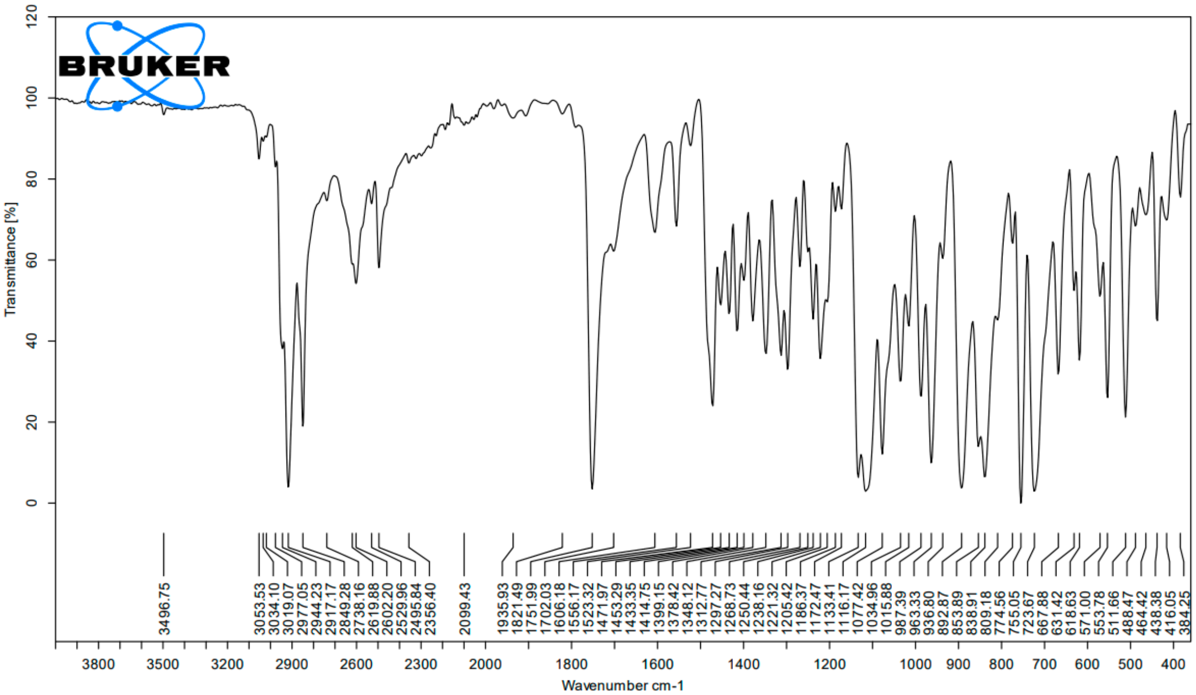

Figure S19. FT-IR spectrum of OE05.

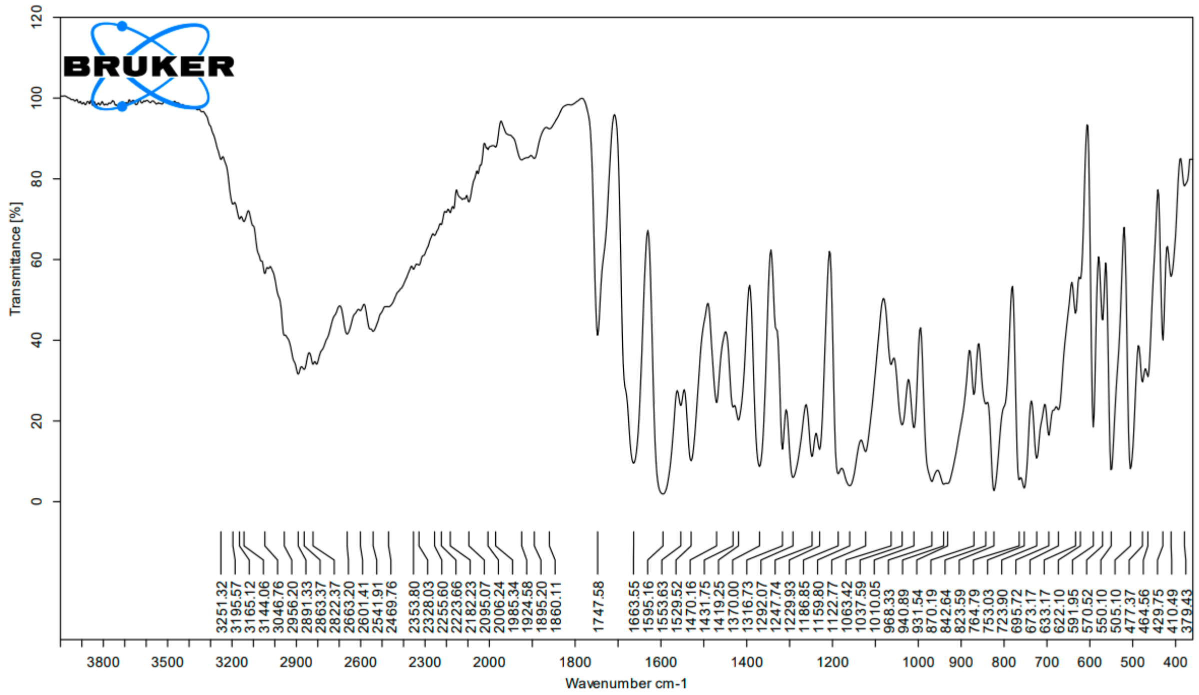

Figure S20. FT-IR spectrum of OE06.

4. Absorption spectra

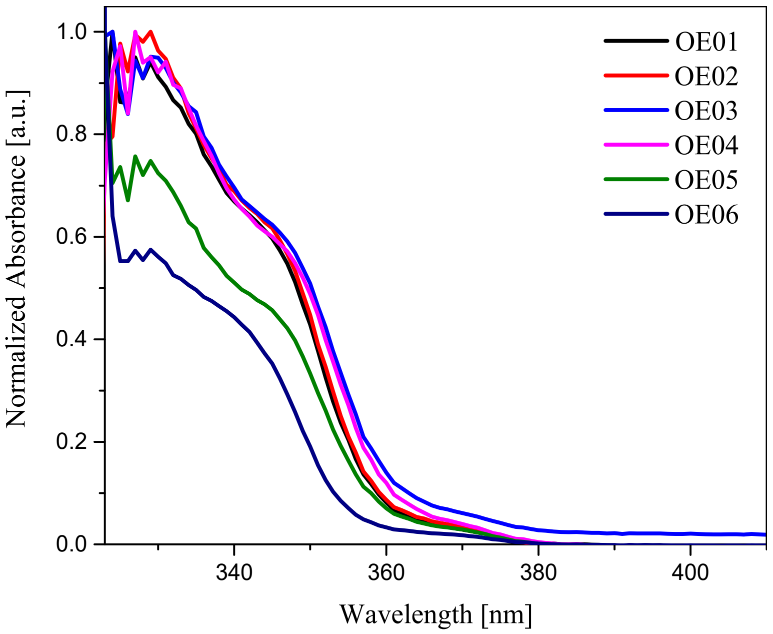

Figure S21. Normalized absorption spectra of oxime esters in acetone (ACE) recorded at room temperature.

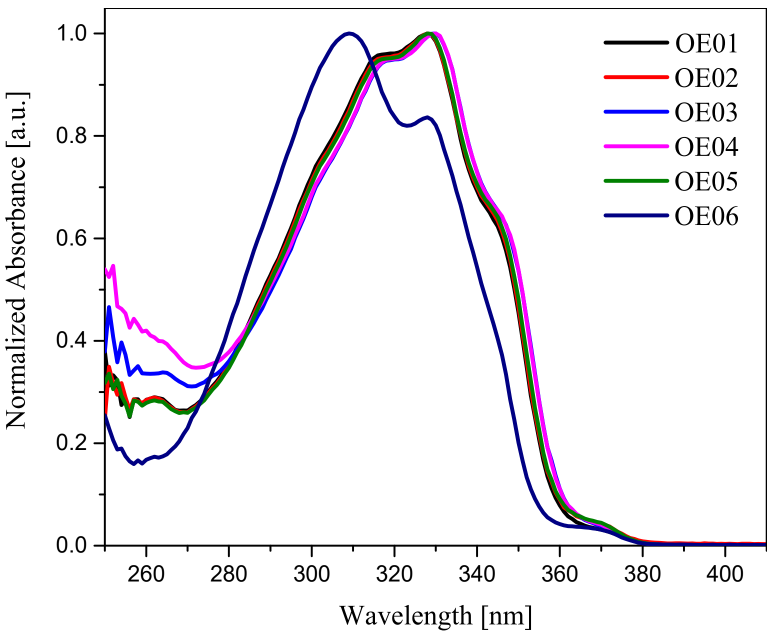

Figure S22. Normalized absorption spectra of oxime esters in ethyl acetate (AcOEt) recorded at room temperature.

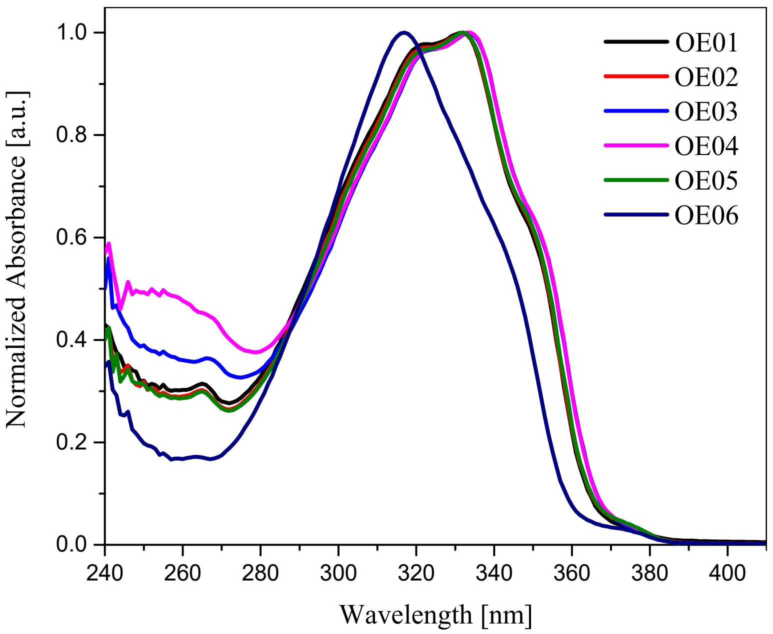

Figure S23. Normalized absorption spectra of oxime esters in chloroform ($CHCl_3$) recorded at room temperature.

https://doi.org/10.3390/ma19030558

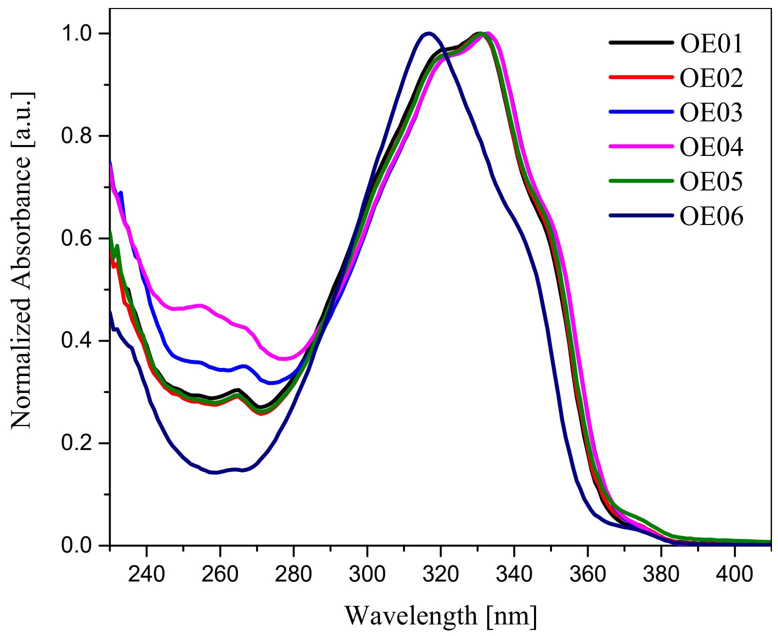

Figure S24. Normalized absorption spectra of oxime esters in dichloromethane (DCM) recorded at room temperature.

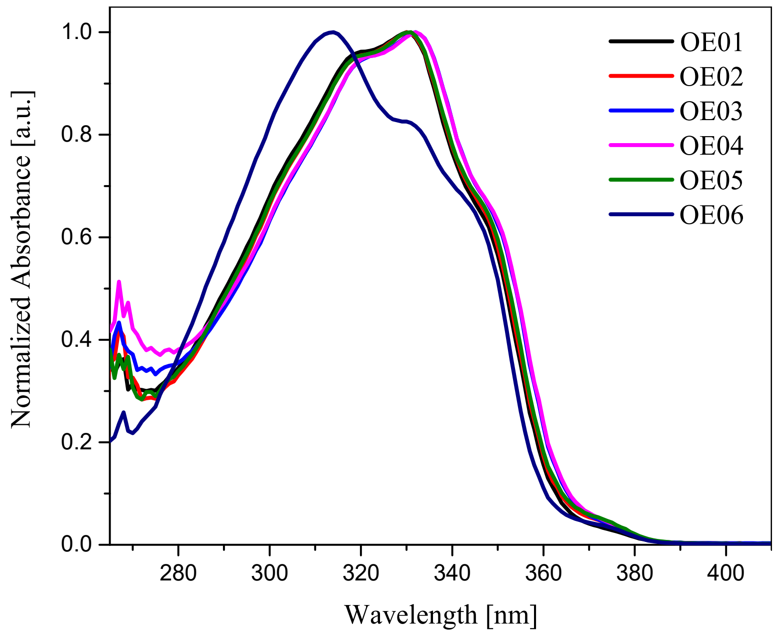

Figure S25. Normalized absorption spectra of six oxime esters in *N,N*-dimethylformamide (DMF) recorded at room temperature.

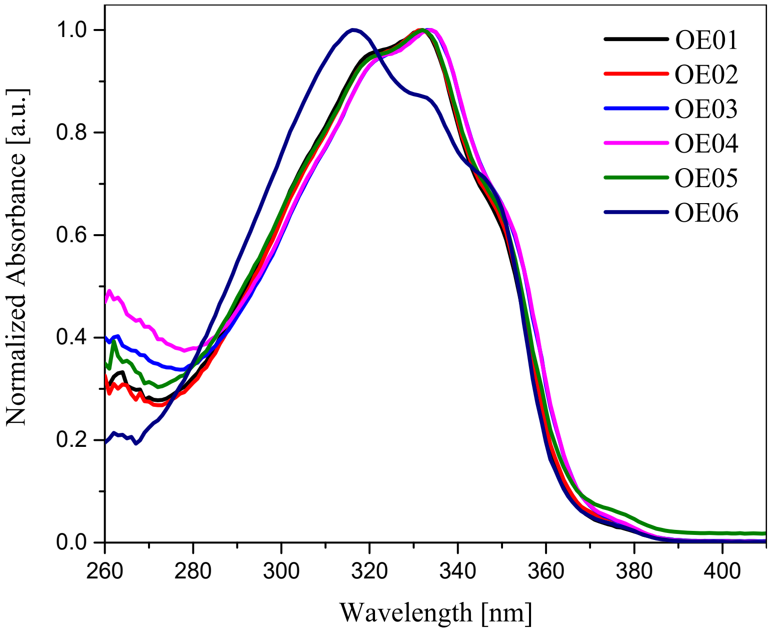

Figure S26. Normalized absorption spectra of oxime esters in dimethyl sulfoxide (DMSO) recorded at room temperature.

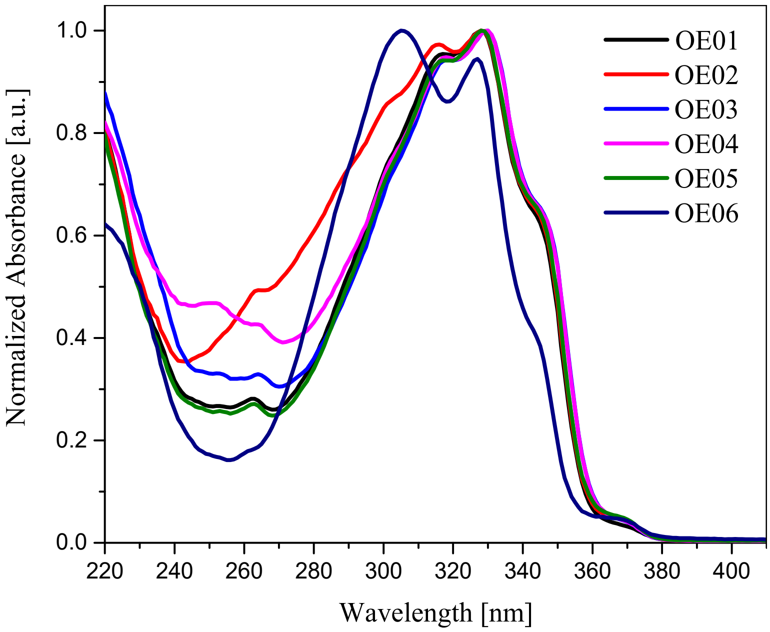

Figure S27. Normalized absorption spectra of oxime esters in diethyl ether (Et_2O) recorded at room temperature.

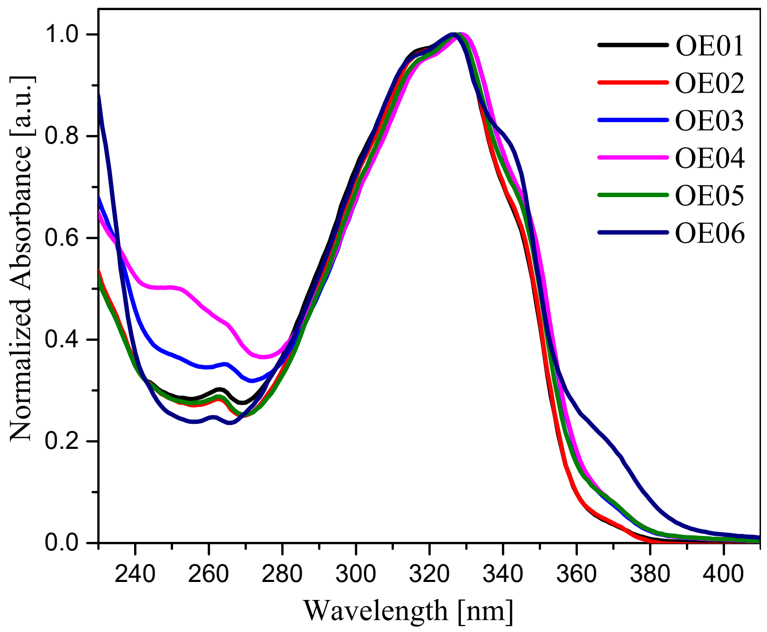

Figure S28. Normalized absorption spectra of oxime esters in acetonitryle (MeCN) recorded at room temperature.

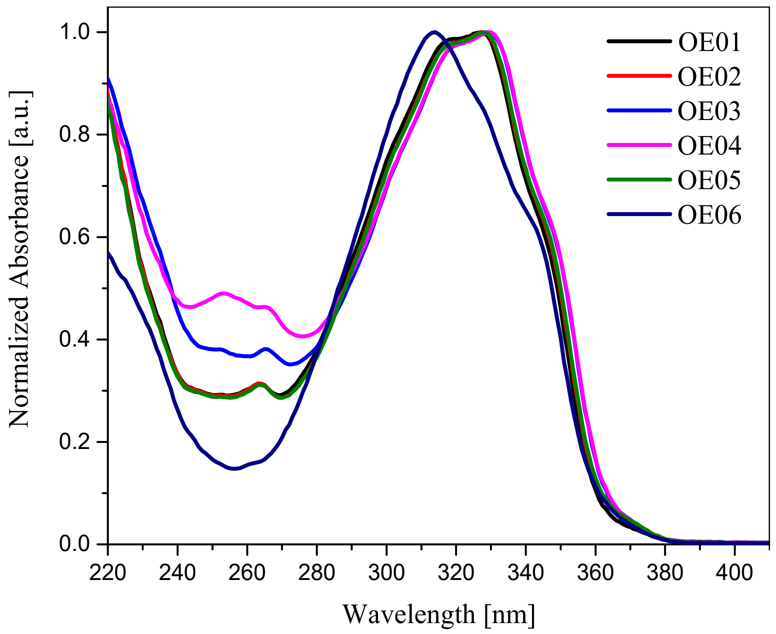

Figure S29. Normalized absorption spectra of oxime esters in methanol (MeOH) recorded at room temperature.

https://doi.org/10.3390/ma19030558

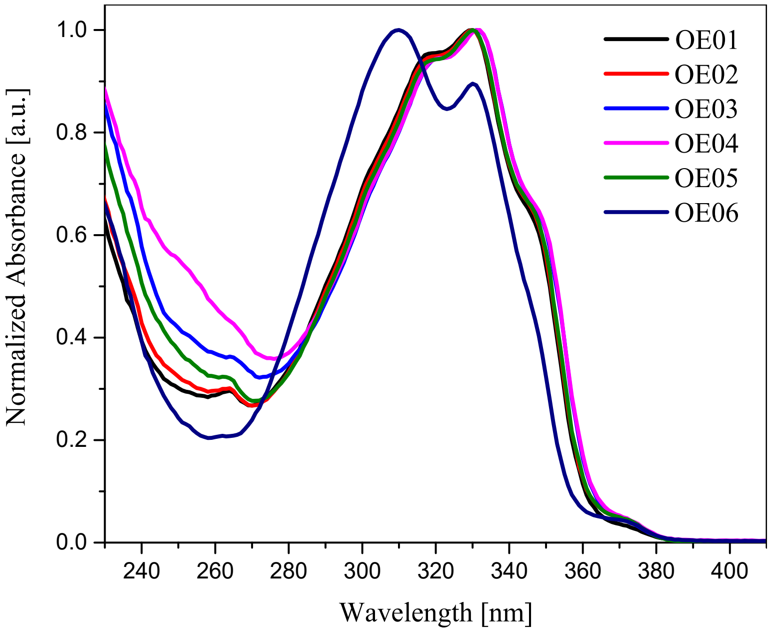

Figure S30. Normalized absorption spectra of oxime esters in tetrahydrofuran (THF) recorded at room temperature.

5. Fluorescence spectra

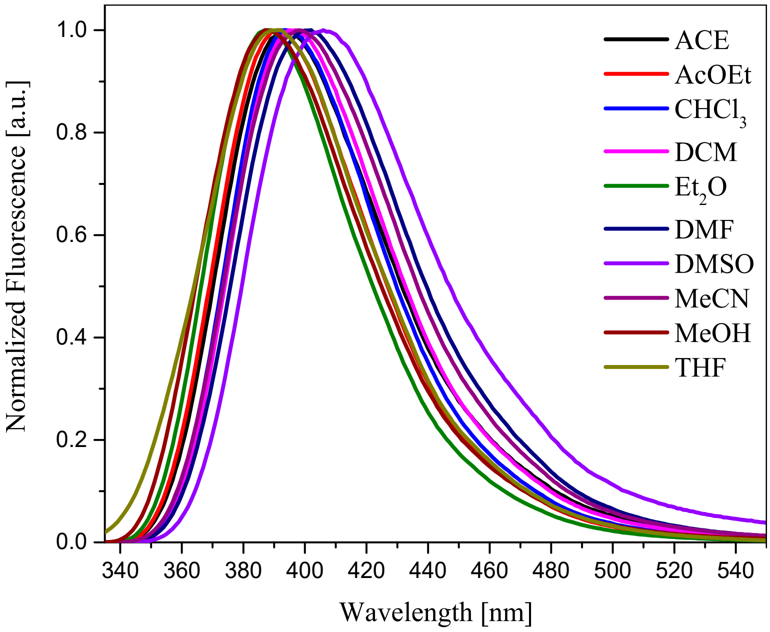

Figure S31. Normalized fluorescence spectra of OE01 in different solvents recorded at room temperature.

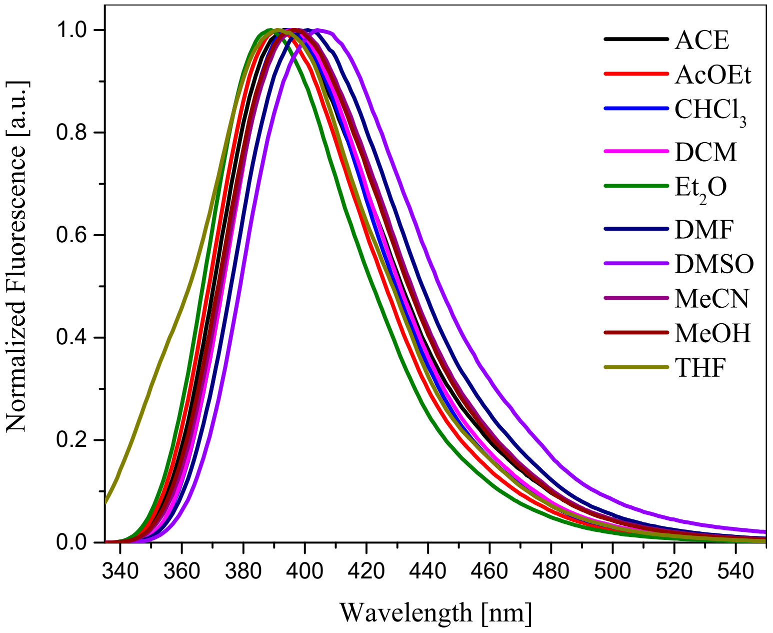

Figure S32. Normalized fluorescence spectra of OE02 in different solvents recorded at room temperature.

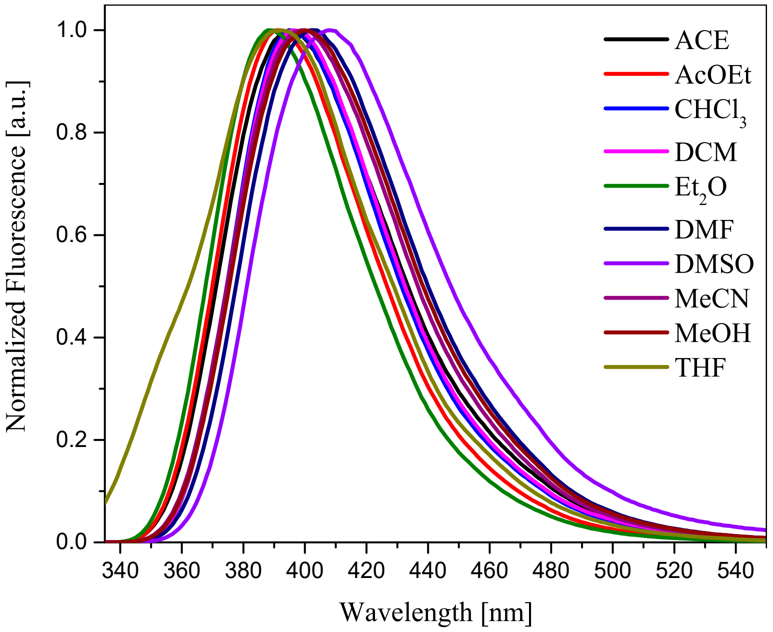

Figure S33. Normalized fluorescence spectra of OE03 in different solvents recorded at room temperature.

https://doi.org/10.3390/ma19030558

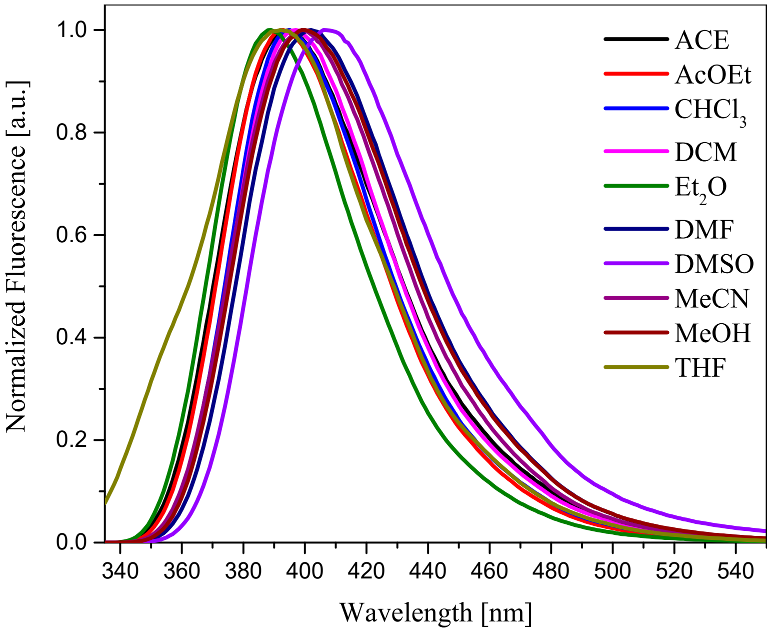

Figure S34. Normalized fluorescence spectra of OE04 in different solvents recorded at room temperature.

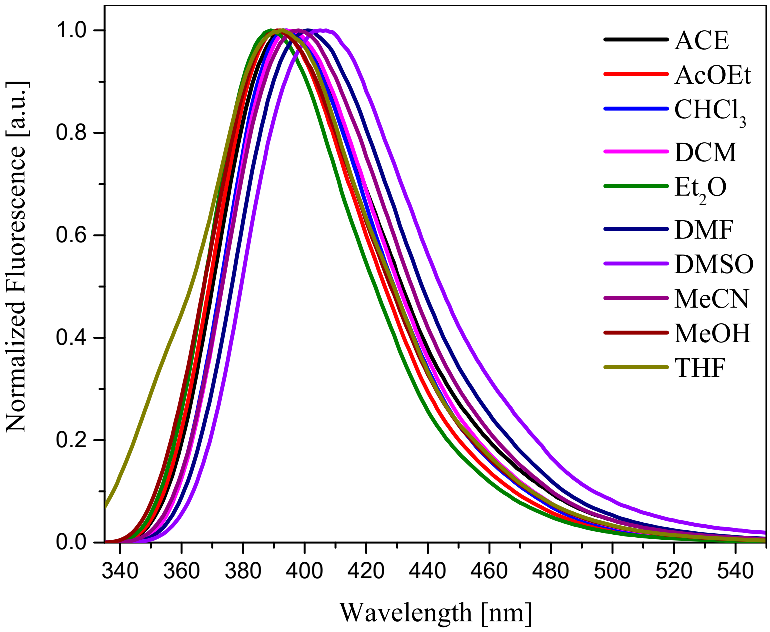

Figure S35. Normalized fluorescence spectra of OE05 in different solvents recorded at room temperature.

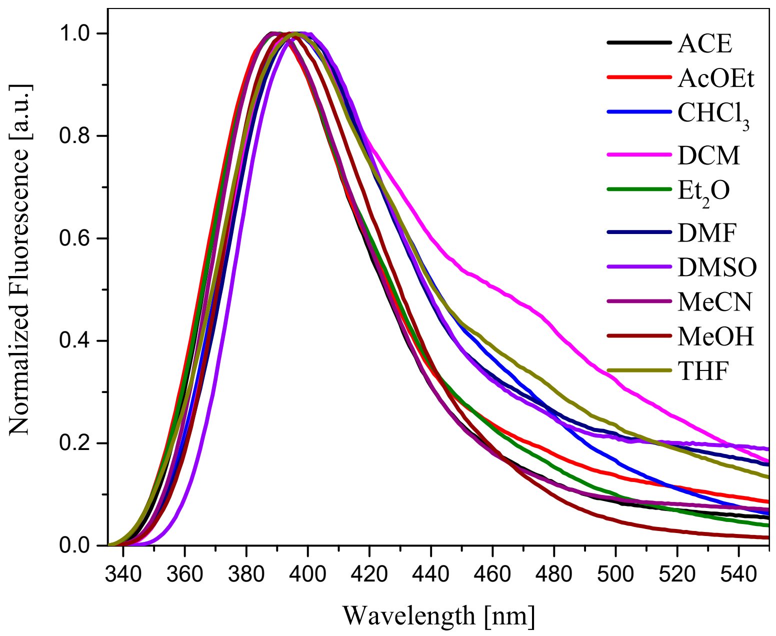

Figure S36. Normalized fluorescence spectra of OE06 in different solvents recorded at room temperature.

5. Photolysis of oxime esters in acetonitrile

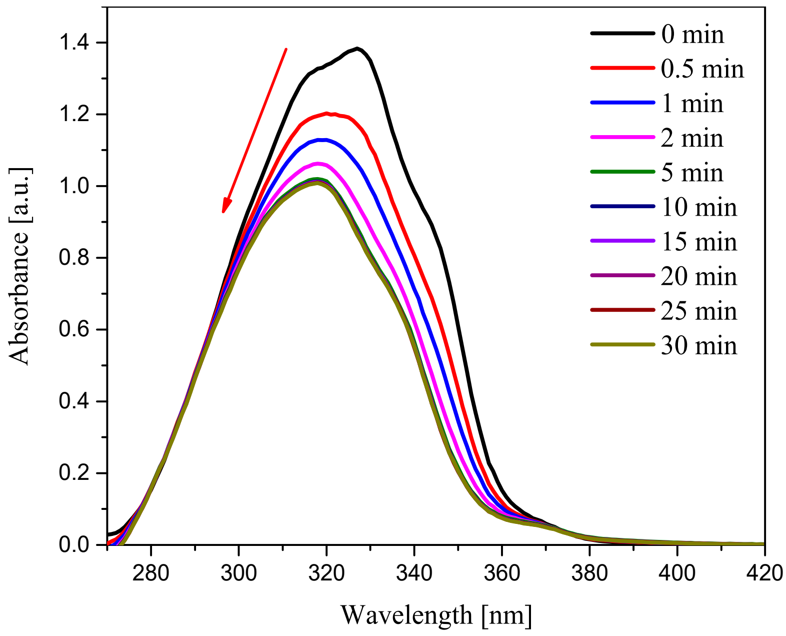

Figure S37. Photolysis of OE01 in MeCN under @LED 365 nm with a light intensity (50 mW/cm²).

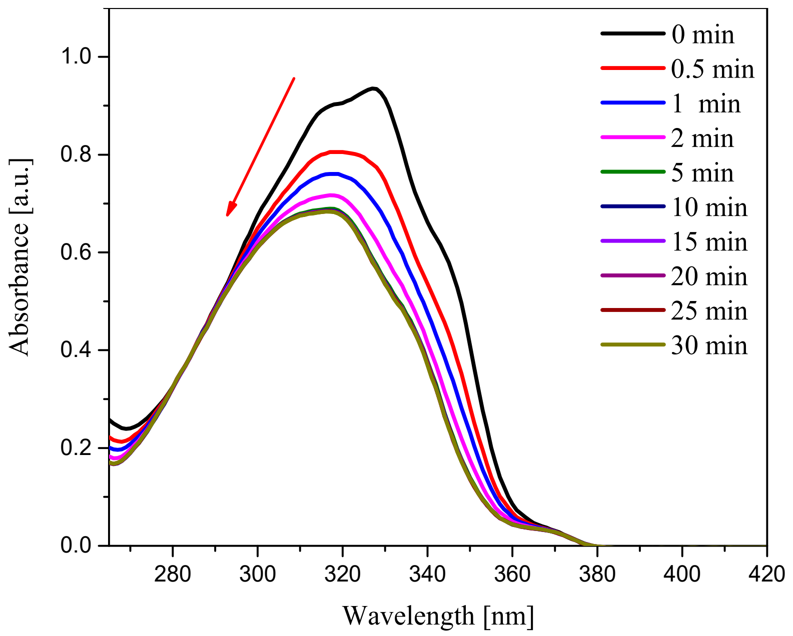

Figure S38. Photolysis of OE02 in MeCN under @LED 365 nm with a light intensity (50 mW/cm²).

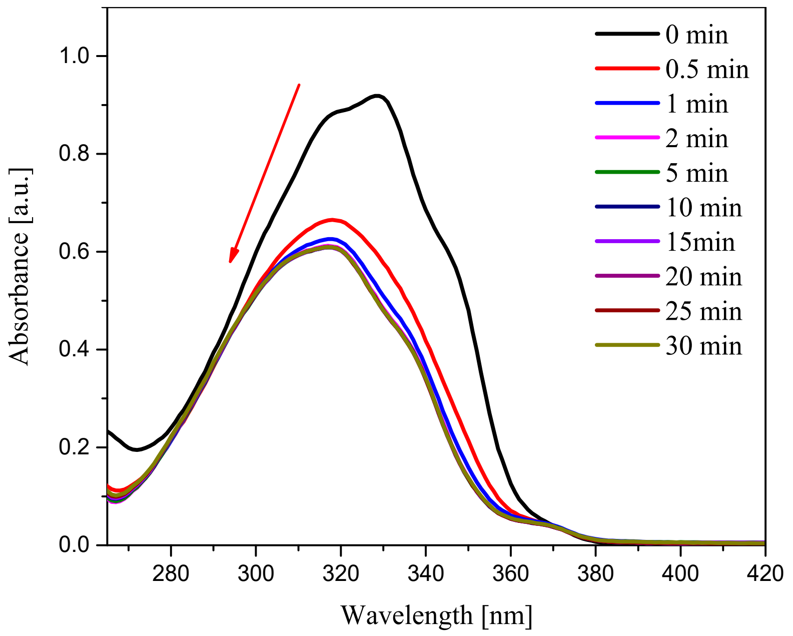

Figure S39. Photolysis of OE03 in MeCN under @LED 365 nm with a light intensity (50 mW/cm²).

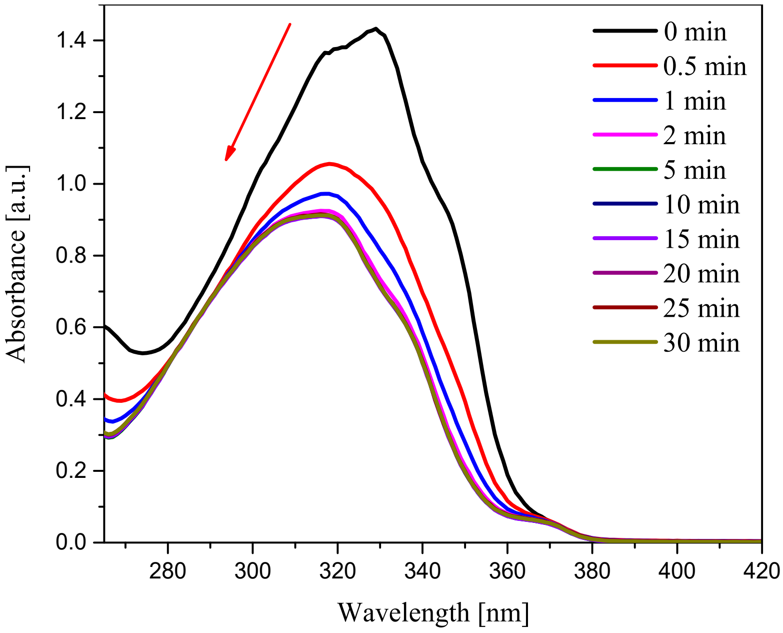

Figure S40. Photolysis of OE04 in MeCN under @LED 365 nm with a light intensity (50 mW/cm²).

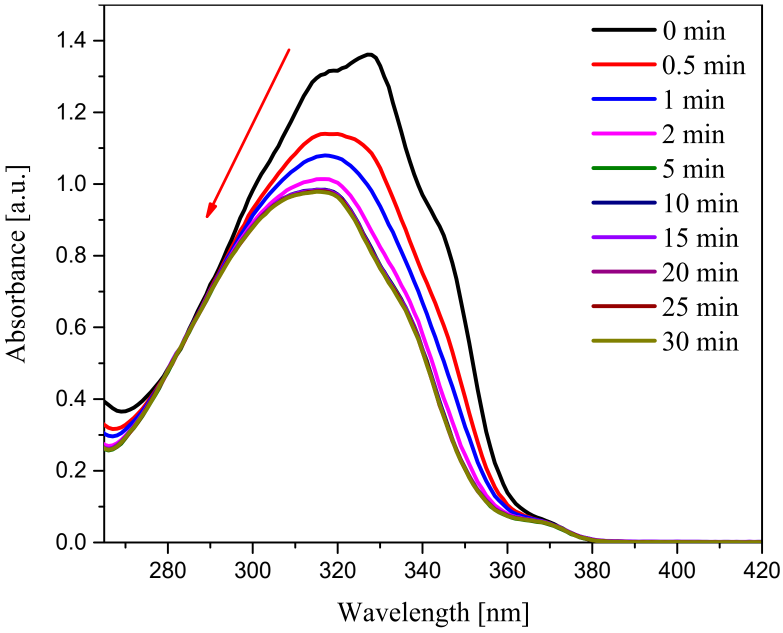

Figure S41. Photolysis of OE05 in MeCN under @LED 365 nm with a light intensity (50 mW/cm²).

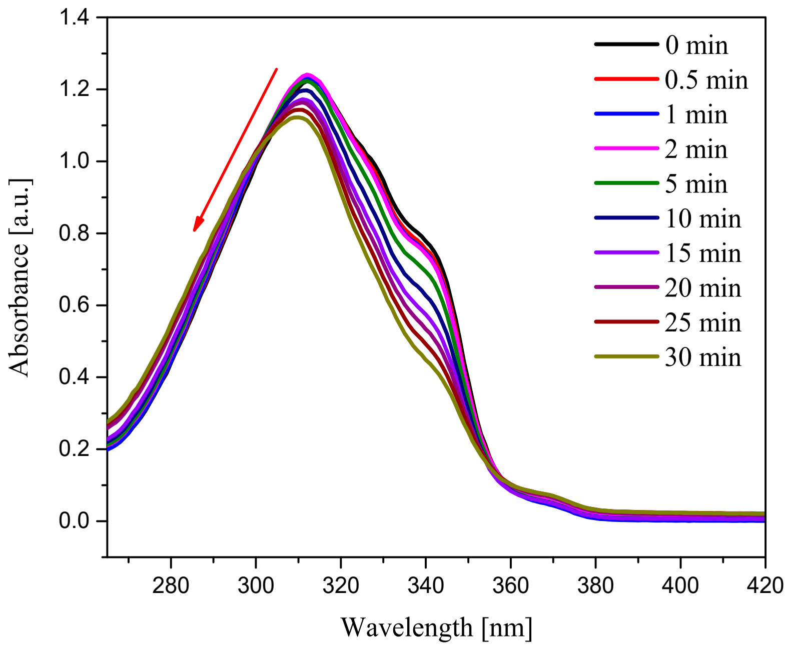

Figure S42. Photolysis of OE06 in MeCN under @LED 365 nm with a light intensity (50 mW/cm²).

References

1. Zhong, X.; Yang, Q.; Chen, Y.; Jiang, Y.; Dai, Z. Aggregation-induced fluorescence probe for hypochlorite imaging in mitochondria of living cells and zebrafish. *J Mater Chem B* **2020**;8:7375-81. https://doi.org/10.1039/D0TB01496F.
2. Wang, G.; Peng, Z.; Li, Y.; Gong, Z.; Ma, X. Benzothiazole-oxazole type alpha-glucosidase inhibitor and preparation method and application thereof, Patent CN 108530438A. Publication date 2018-09-14.
3. Karakurt, A.; Alagöz, M.A.; Sayoğlu, B.; Çalış, Ü.; Dalkara, S. Synthesis of some novel 1-(2-naphthyl)-2-(imidazol-1-yl) ethanone oxime ester derivatives and evaluation of their anticonvulsant activity. *Eur J Med Chem* **2012**;57:275-82. https://doi.org/10.1016/j.ejmech.2012.08.037.

Disclaimer/Publisher's Note: The statements, opinions and data contained in all publications are solely those of the individual author(s) and contributor(s) and not of MDPI and/or the editor(s). MDPI and/or the editor(s) disclaim responsibility for any injury to people or property resulting from any ideas, methods, instructions or products referred to in the content.
